# Low dose dimethyl sulfoxide driven gross molecular changes have the potential to interfere with various cellular processes

**DOI:** 10.1038/s41598-018-33234-z

**Published:** 2018-10-04

**Authors:** Sinem Tunçer, Rafig Gurbanov, Ilir Sheraj, Ege Solel, Okan Esenturk, Sreeparna Banerjee

**Affiliations:** 1 Department of Biological Sciences, Orta Dogu Teknik Universitesi (ODTU/METU), Ankara, 06800 Turkey; 2 Department of Molecular Biology and Genetics, Bilecik Şeyh Edebali University, Bilecik, 11230 Turkey; 3 Department of Chemistry, Orta Dogu Teknik Universitesi (ODTU/METU), Ankara, 06800 Turkey; 4 Present Address: Vocational School of Health Services, Department of Medical Laboratory Techniques, Bilecik Şeyh Edebali University, Bilecik, 11230 Turkey; 5 Present Address: Department of Biomedicine, University of Bergen, Postbox 7804, Bergen, N-5020 Norway

## Abstract

Dimethyl sulfoxide (DMSO) is a small molecule with polar, aprotic and amphiphilic properties. It serves as a solvent for many polar and nonpolar molecules and continues to be one of the most used solvents (vehicle) in medical applications and scientific research. To better understand the cellular effects of DMSO within the concentration range commonly used as a vehicle (0.1–1.5%, v/v) for cellular treatments, we applied Attenuated Total Reflectance (ATR) Fourier Transform Infrared (FT-IR) spectroscopy to DMSO treated and untreated epithelial colon cancer cells. Both unsupervised (Principal Component Analysis-PCA) and supervised (Linear Discriminant Analysis-LDA) pattern recognition/modelling algorithms applied to the IR data revealed total segregation and prominent differences between DMSO treated and untreated cells at whole, lipid and nucleic acid regions. Several of these data were supported by other independent techniques. Further IR data analyses of macromolecular profile indicated comprehensive alterations especially in proteins and nucleic acids. Protein secondary structure analysis showed predominance of β-sheet over α-helix in DMSO treated cells. We also observed for the first time, a reduction in nucleic acid level upon DMSO treatment accompanied by the formation of Z-DNA. Molecular docking and binding free energy studies indicated a stabilization of Z-DNA in the presence of DMSO. This alternate DNA form may be related with the specific actions of DMSO on gene expression, differentiation, and epigenetic alterations. Using analytical tools combined with molecular and cellular biology techniques, our data indicate that even at very low concentrations, DMSO induces a number of changes in all macromolecules, which may affect experimental outcomes where DMSO is used as a solvent.

## Introduction

Dimethyl sulfoxide (DMSO; C_2_H_6_OS) is a small amphipathic organic molecule with a hydrophilic sulfoxide group and two hydrophobic methyl groups. Being also an aprotic, DMSO tends to accept rather than donate protons. It can solubilize a wide variety of organic and inorganic compounds at high concentrations. This, as well as its apparent low toxicity, has made DMSO to be accepted as a “universal solvent” which is widely used as a vehicle in scientific research, drug screening settings and biomedical applications. DMSO is also a commonly used cryoprotectant to protect cells from ice crystal-induced mechanical injury^1–3^.

Several studies have reported that DMSO plays multiple roles in cellular functions such as inflammation, lipid metabolism, apoptosis, cell cycle, protein expression, differentiation, molecule binding, enzyme activity, reactive oxygen species scavenging, cell polarization, radioprotection, and autophagy^4–6^. Based on the multitude effects of DMSO reported in the literature, we aimed to examine systematically the global effects as well as individual changes in macromolecules in epithelial cells treated with low concentrations of DMSO (0.1–1.5%, v/v). This is the first study demonstrating that DMSO induced a number of gross biomolecular changes in all macromolecules (proteins, lipids and nucleic acids), which may influence experimental results where DMSO is used as a solvent.

## Results and Discussion

### Growth inhibition and reduced ROS formation observed in cells treated with DMSO

**Figure 1:**
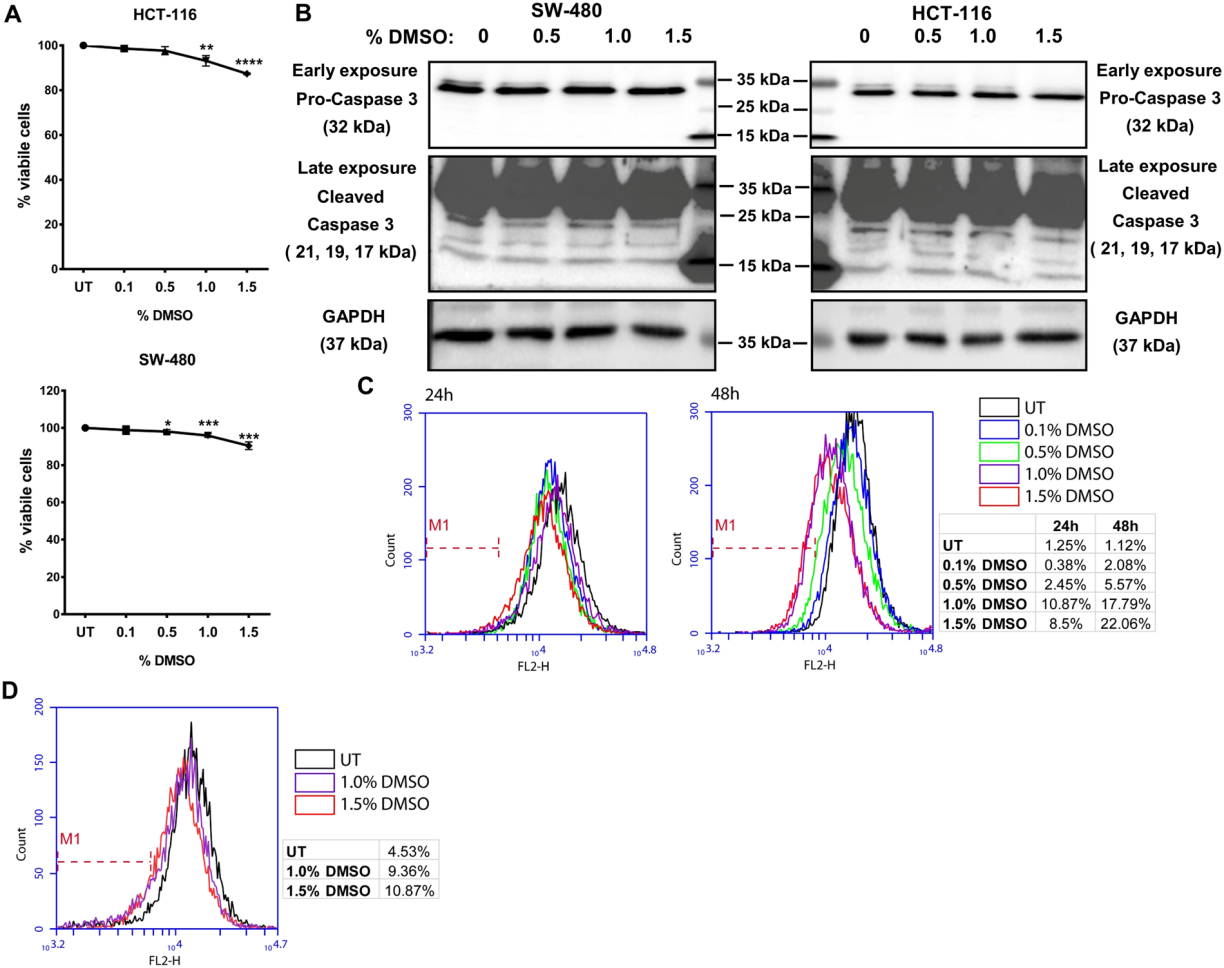
DMSO shows growth inhibitory and ROS reducing effects in HCT-116 and SW-480 cells. (**A**) After 24 h of incubation with the indicated doses of DMSO, cellular growth was investigated in HCT-116 and SW-480 cells with the MTT assay. The effect of DMSO treatment on cellular growth is expressed as percent viability with respect to untreated (UT) cells. The results from three independent replicates each with eight technical replicates are given as mean ± SEM. t test was used to analyze the results. (**B**) Formation of cleaved Caspase 3 in DMSO treated cells was investigated by western blot. GAPDH was used as loading control. (**C**) DHE assay was used to measure intercellular ROS levels in DMSO treated HCT-116 (24 h and 48 h), and (**D**) SW-480 cells (24 h). M1 gate was set on the basis of UT cells.

Colorectal cancer (CRC) cell lines HCT-116 and SW-480 that have an epithelial phenotype were incubated with different concentrations of DMSO for 24 h and the effect on cellular growth was investigated for both cell lines with an MTT assay. As seen in Fig. 1A, DMSO showed a dose dependent effect on cell proliferation; cells treated with 1.5% DMSO showed an approximately 10% reduction in cell growth. However, reduction in cell growth was not due to the induction of apoptosis as we did not observe any Caspase 3 activation in the cells treated with the same doses of DMSO (Fig. 1B). A 10% reduction in cell growth was seen in 0.5% DMSO treated MCF-10A, a non-tumorigenic normal breast epithelial cell line (Supplementary Fig. S1).

Since several previous studies have shown that DMSO has antioxidant properties^2^, we wanted to determine whether low doses of DMSO had any effect on cellular Reactive Oxygen Species (ROS). For this, we used Dihydroethidium (DHE), a cell-permeable fluorescent dye that exhibits blue fluorescence in the cytosol until oxidized. The oxidized DHE intercalates with the cell’s DNA, staining the nucleus a bright fluorescent red that can be assayed. DHE has been shown to be oxidized by superoxide to form 2-hydroxyethidium (2-OH-E^+^) or by non-specific oxidation by other sources of ROS to form ethidium (E^+^)^7,8^. As shown in Fig. 1C,D, treatment of both HCT-116 and SW-480 cells with low doses of DMSO for 48 h reduced cellular ROS level in a dose dependent manner, with the antioxidant effect more prominent in HCT-116 compared to SW-480 cells (Fig. 1C).

### IR spectroscopy coupled with PCA and LDA display a clear distinction between DMSO treated and untreated cells

In order to have a more macroscopic understanding of the effects of low dose treatment of cells with DMSO, we carried out a Fourier Transform IR (FT-IR) spectroscopic analysis. To this effect, we first established whether, based on spectral data, DMSO treated cells could be distinguished from the untreated (UT) cells using Principal Component Analysis (PCA). PCA is an exploratory and unsupervised pattern recognition method analysing the natural relationship of samples without any bias. Accordingly, the samples are unpredictably sorted in several different categories beyond their primary quality and grouping positions^9^.

**Figure 2:**
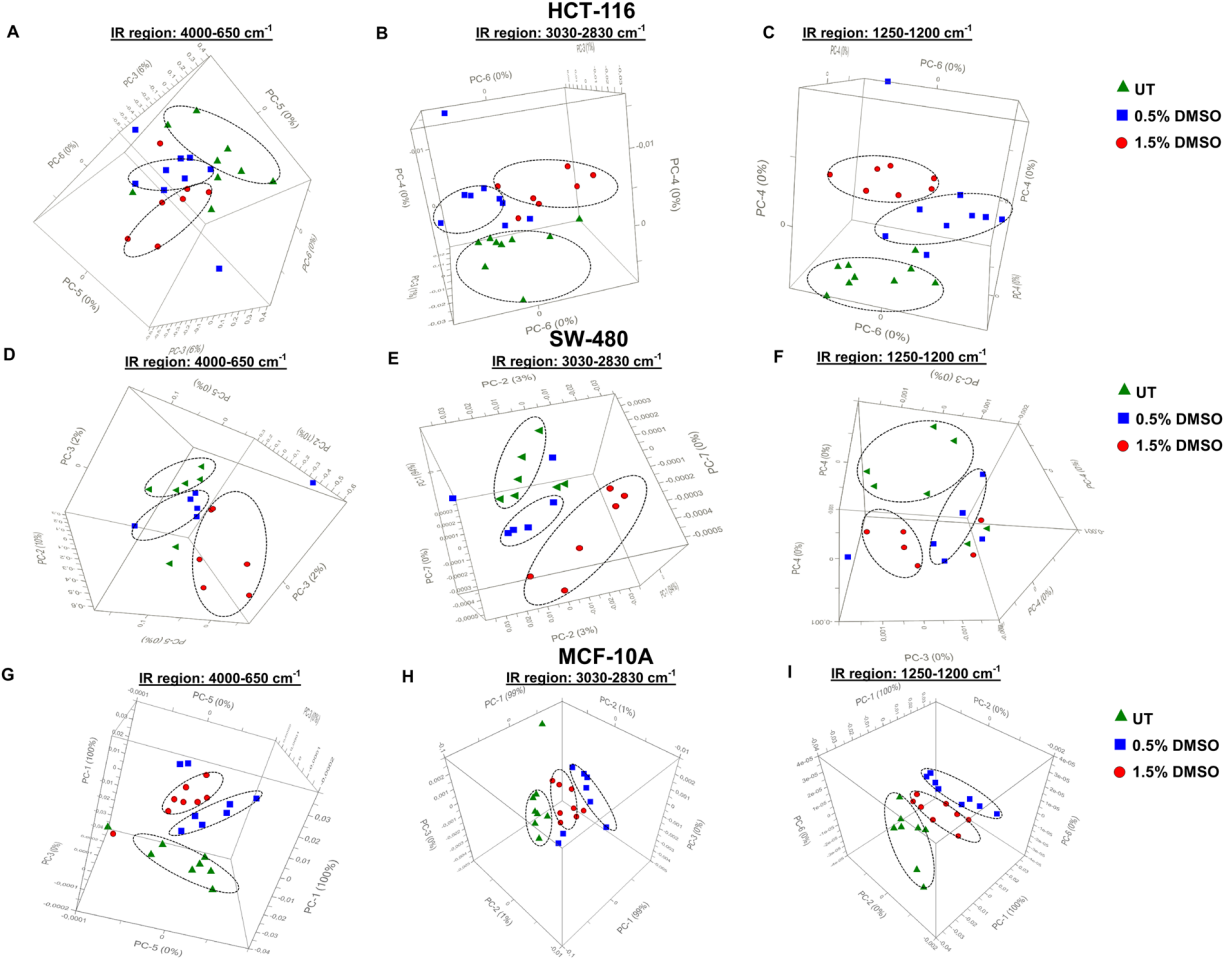
Principal Components Analysis (PCA) 3D scores plots for DMSO treated and untreated (UT) HCT-116, SW-480 and MCF-10A cells. PCA 3D scores plots for DMSO treated (0.5% and 1.5% v/v) cells and untreated (UT) HCT-116 (**A–C**), SW-480 (**D–F**) and MCF-10A (**G–I**) cells in the 4000–650 cm^−1^, 3030–2830 cm^−1^ and 1250–1200 cm^−1^ spectral regions.

Figure 2 shows the 3D scores plots for DMSO-treated and untreated (UT) HCT-116, SW-480, and MCF-10A cells in the whole (4000-650 cm^−1^), lipid (3030–2800 cm^−1^) and nucleic acid (1250–1200 cm^−1^) IR spectral regions. Figure 2A,D and G represent clear differentiation of 0.5% and 1.5% DMSO treated groups from each other and from UT groups in whole spectral region for HCT−116, SW-480, and MCF-10A, respectively. A clear segregation was similarly observed in the lipid (Fig. 2B,E,H) and nucleic acid (Fig. 2C,F,I) regions for all three cell lines. The well-segregated scores plots in different spectral regions indicate remarkable dose-dependent molecular alterations induced by DMSO. Supervised Linear Discriminant Analysis (LDA) results in the whole spectral region further confirmed these findings (Supplementary Figs S2–4, for HCT-116, SW-480, and MCF-10A, respectively). As shown in LDA discrimination plots, and prediction and confusion matrices, 0.5% and 1.5% DMSO treated groups could be clearly discriminated from each other and from UT cells with 100% accuracy.

Treatment of cells with different doses of DMSO generated unique fingerprint IR spectra (1800–650 cm^−1^) in HCT-116 cells confirming the occurrence of major molecular alterations (Supplementary Fig. S5). The spectrum consists of hundred or more bands, and it is not necessary to allocate most of them^10^. To get a better understanding of the qualitative and quantitative alterations occurring in individual macromolecules in the presence of DMSO, we analysed the positions of individual spectral bands and their intensities emerging from different functional groups of macromolecules. Of note, wavenumber and intensity of IR bands correspond to structural alterations and concentration of specific molecule, respectively^10,11^.

### DMSO decreases total nucleic acid content and delays cell cycle progression

**Figure 3:**
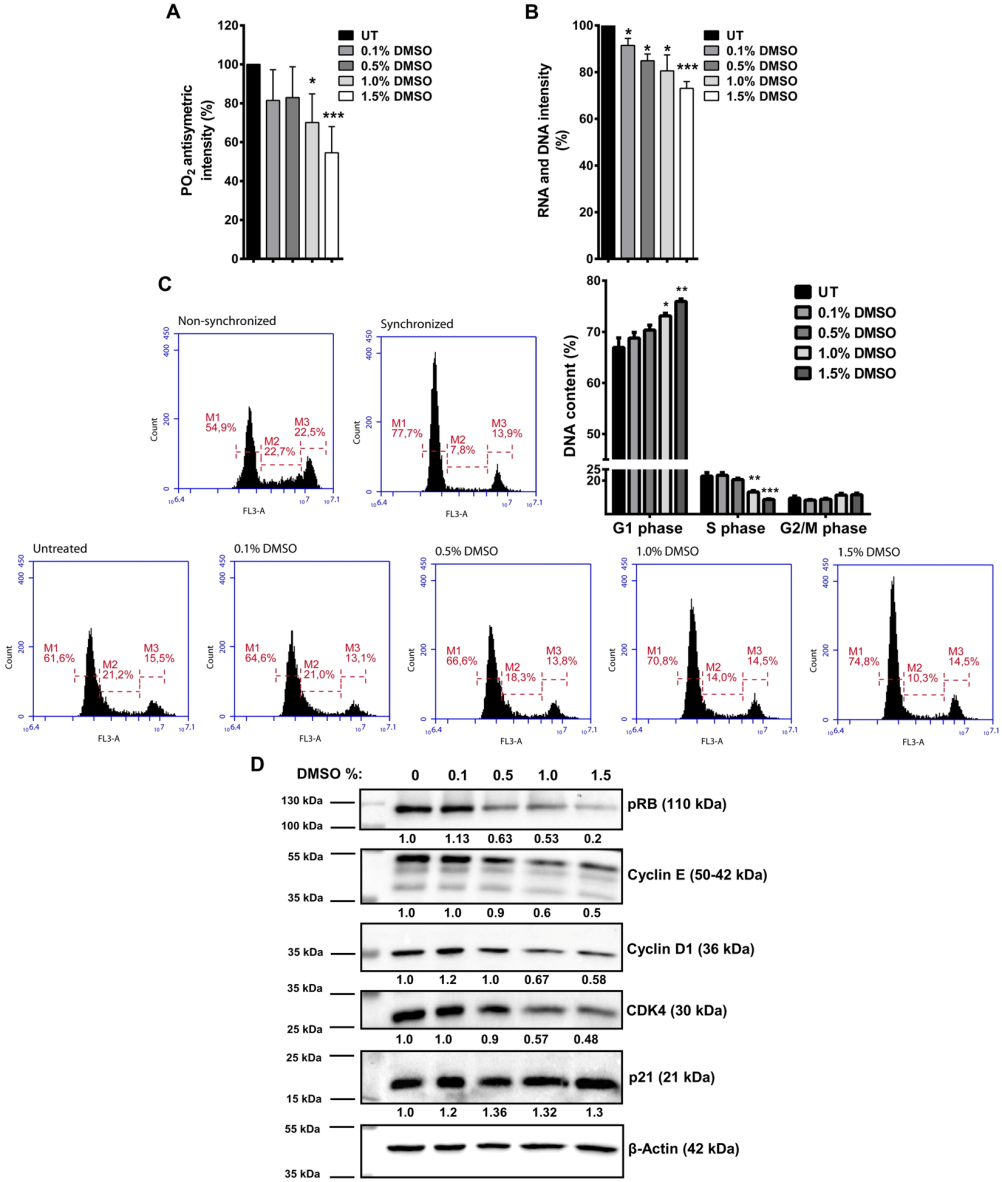
DMSO treatment alters cellular nucleic acid content in HCT-116 cells. (**A**) PO_2_ antisymmetric band intensity (%) values, and (**B**) Ribose ring vibrations band intensity (%) values for DMSO treated and untreated (UT) HCT-116 cells are shown. The results are given as mean ± SEM. t test was used to compare DMSO treated cells with UT cells. (**C**) HCT-116 cells were synchronized by serum starvation for 24 h. Following starvation, cells were treated with serum containing medium in the presence or absence (untreated-UT) of DMSO for 7 h. Representative histograms for cell cycle phase distributions of DMSO treated and untreated cells after release, and non-starved (non-synchronized) and starved (synchronized) cells are given. Percentages of cell cycle phases are given as mean ± SEM of three independent biological replicates each with two technical replicates. Statistical significance was calculated with respect to UT cells by using t test. (**D**) After 7 h release following 24 h starvation, expression levels of cell cycle proteins associated with G1 to S phase transition were examined in DMSO treated or untreated cells by western blot. β-Actin was used as loading control for normalization. Changes in the expression levels are given as fold change with respect to untreated cells.

To determine any alterations in nucleic acid content induced by low doses of DMSO, we analysed the PO_2_ antisymmetric band from the absorbance spectrum located in between 1242–1238 cm^−1^ that is assigned to total nucleic acids^12^. Additionally, the band located at 915 cm^−1^ and emerging from Ribose ring vibrations of total RNA and DNA^13,14^ was analysed from the second derivative spectra. The intensities (%) of the PO_2_ antisymmetric band are summarized in Fig. 3A for HCT-116 cells. We observed a dose-dependent decrease in the intensity of this band in the presence of DMSO. This decrease was statistically significant in the 1.0% and 1.5% DMSO-treated cells with respect to the control (UT) cells (Fig. 3A). The intensities (%) of total RNA and DNA bands were statistically significantly reduced in a dose dependent manner in DMSO treated HCT-116 cells at all doses compared to the control cells (Fig. 3B). Similar results were obtained for these bands in SW-480 cells (Supplementary Fig. S6A). On the other hand, when compared to HCT-116 and SW-480 CRC cells, a more pronounced decrease in the percentage of total nucleic acids was seen in the DMSO treated non-tumorigenic MCF-10A cells (Supplementary Fig. S6B).

Cells incubated with DMSO in the range of 1.0–2.0%, v/v have been reported to exhibit cellular differentiation accompanied by growth inhibition and arrest at the G1 phase of the cell cycle^15–18^. Since the PO_2_ antisymmetric and Ribose ring vibrations of total RNA and DNA band intensities revealed a decrease in nucleic acid content in DMSO treated cells, we investigated the effect of DMSO on cell cycle progression. We observed that even at the low dose of 0.5% (v/v), DMSO treatment resulted in the accumulation of cells at the G1 phase of the cell cycle (Supplementary Fig. S7A). This effect of DMSO was more prominent when the cells were synchronized through serum starvation and released. 7 h after release, the percentages of the cells at the G1/S boundary of the cell cycle in DMSO treated cells was higher (Fig. 3C), along with an accumulation of p21, and decreased expression of Cyclin E, Cyclin D and Cyclin-Dependent Kinase 4 (CDK4). Additionally, phosphorylation of Retinoblastoma (Rb) was lower in DMSO treated cells compared to control cells (Fig. 3D). Similar results were also observed in SW-480 cells (Supplementary Fig. S7B).

Although we did not address the mechanism through which DMSO treatment delayed the cell cycle progression in HCT-116 and SW-480 cells, we suggest that DMSO mediated decrease in cellular ROS may be responsible for the observed effect. This stems from the fact that mitogenic pathways can be stimulated in the presence of ROS; therefore, the entry of cells into the S phase may be regulated by controlling the activity of CDKs and phosphorylation of Rb protein^19^. Thus, it can be concluded that the growth inhibitory effect observed in DMSO treated HCT-116 and SW-480 cells (Fig. 1A) may have resulted from delayed progression through cell cycle phases.

### DMSO alters DNA topology

**Figure 4:**
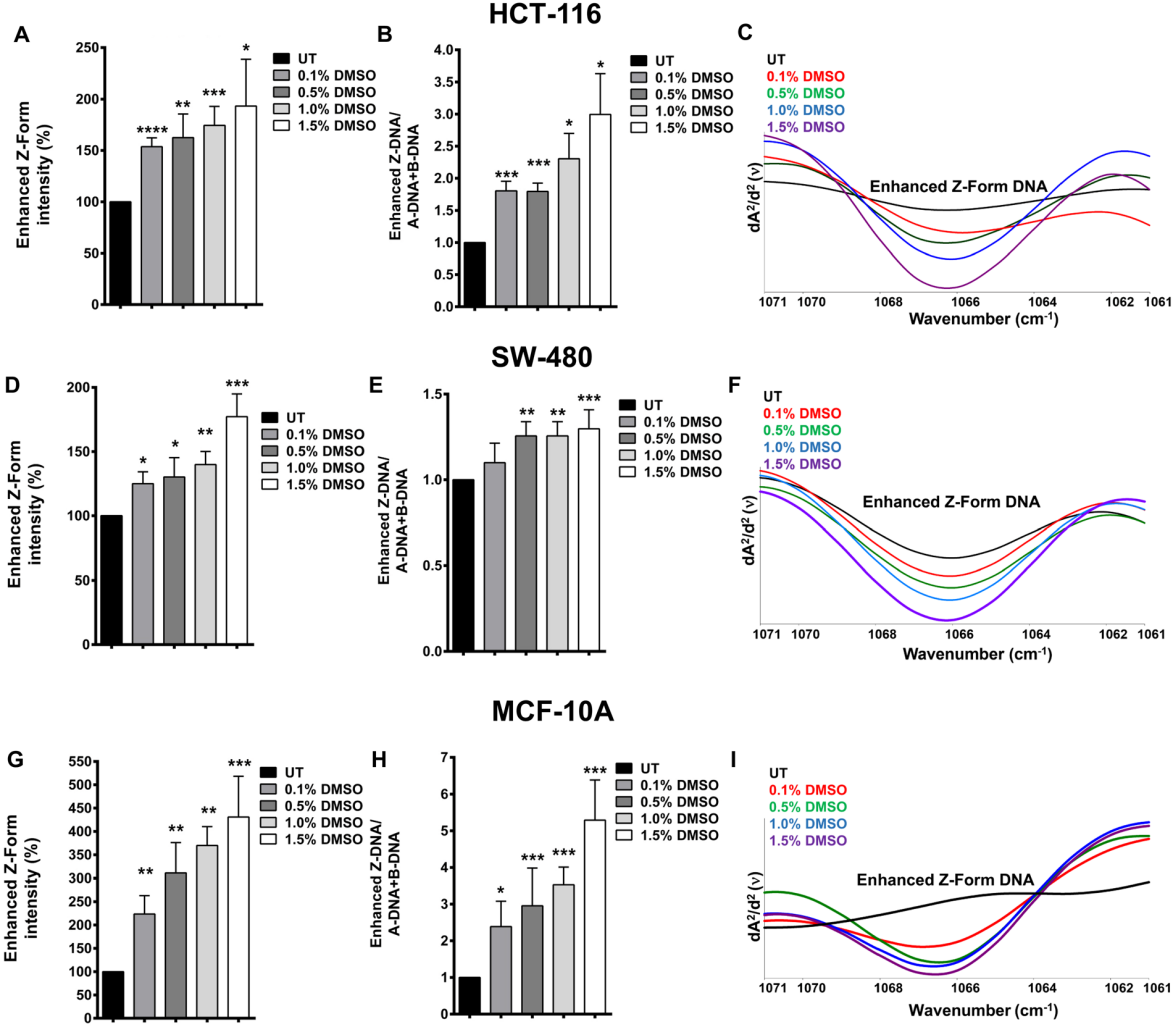
DMSO treatment alters DNA conformation. The % band intensities of enhanced Z-form DNA in DMSO treated and untreated (UT) HCT-116, SW-480, and MCF-10A cells are shown in (**A**,**D** and **G**), respectively. The ratio of the enhanced Z-DNA band intensity to a total of A-DNA and B-DNA forms intensities (The results are given as fold change with respect to UT cells) are given for HCT-116 (**B**), SW-480 (**E**), and MCF-10A (**H**) cells. The representative second derivative and vector-normalized IR spectra of DMSO treated and UT HCT-116 (**C**), SW-480 (**F**), and MCF-10A (**I**) cells are shown in the spectral range of 1071–1061 cm^−1^. The results are given as mean ± SEM. t test was used to compare the results with respect to UT cells.

The sub-bands of PO_2_ antisymmetric band were analysed in detail from the second-derivative IR spectra. These are highly sensitive and marker bands that are assigned to the main B and main A conformations of DNA^12^. The absorbance of the main B and A-forms of DNA are located at 1221 cm^−1^ and 1240 cm^−1^, respectively^20^. Particularly, we analysed the band located at 1066 cm^−1^ that is strongly enhanced specifically in the presence of Z-form DNA^21^. Enhanced Z-form intensities (%) were observed to increase significantly in a dose-dependent manner at all doses of DMSO treatment in all three cell lines analysed in the study: HCT-116 (Fig. 4A,C), SW-480 (Fig. 4D,F), and MCF-10A (Fig. 4G,I). Information about the relative concentration of a specific biomolecule in a system can be achieved by analysing the alterations in band intensity values^22^. When the ratio of the enhanced Z-DNA band intensity to the sum of intensities of A-DNA and B-DNA forms was calculated, we found that the content of Z-DNA increased significantly with DMSO treatment in these cells (Fig. 4B,E,H).

**Figure 5:**
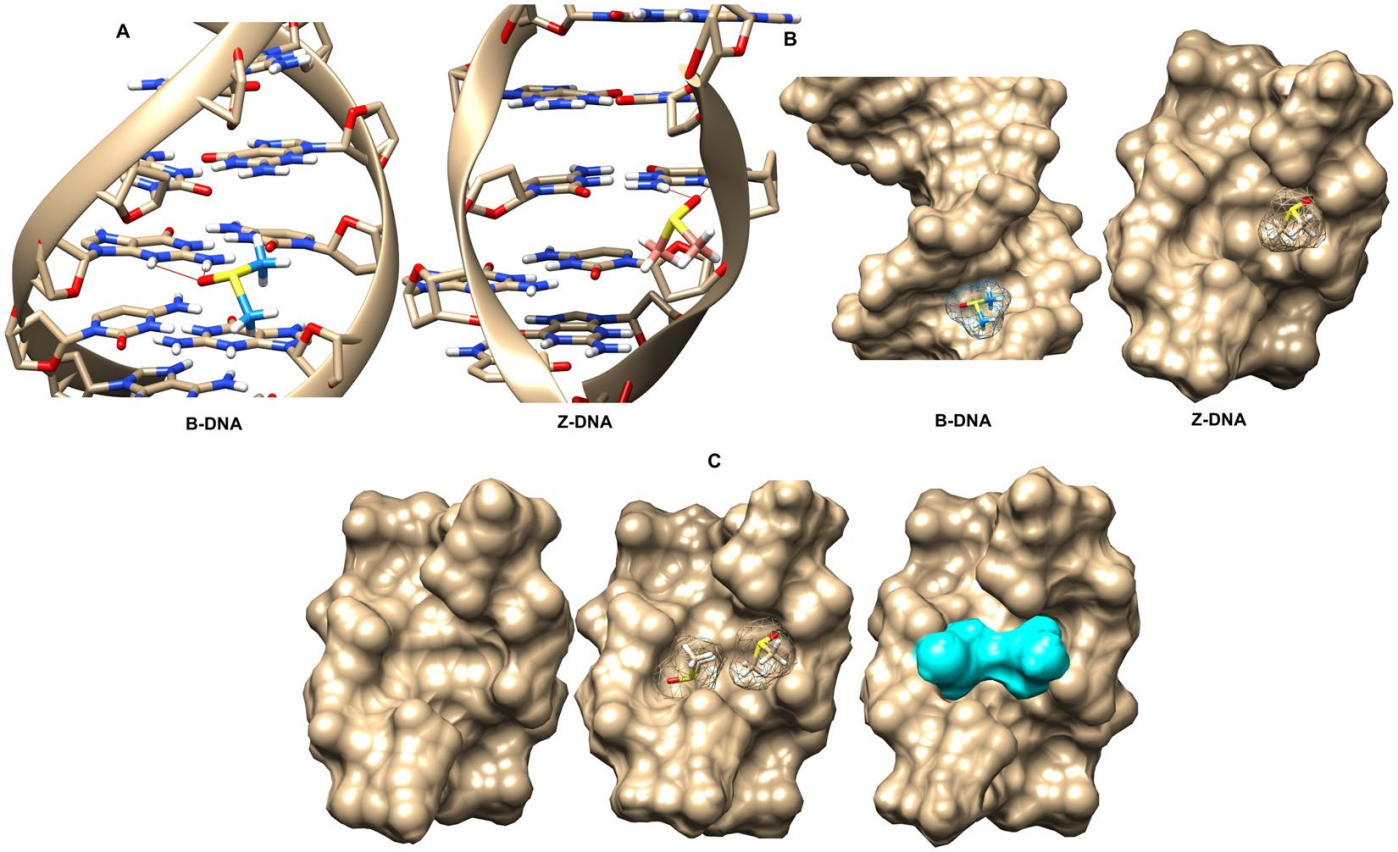
DMSO binding to B-DNA and Z- DNA. (**A**) AutoDock output of DMSO molecule bound to B-DNA and Z-DNA in ball-and-stick representations. (**B**) Solid molecular surface representations of DMSO bound B-DNA and Z-DNA. (**C**) Solid molecular surface representation of Z-DNA and Gaussian output of two DMSO molecules bound to DNA in mesh and solid surface representations, respectively.

*In vivo*, DNA is most frequently found as the canonical duplex right-handed helical structure known as B-DNA^23^. However, DNA is able to fit different conformations other than the familiar right-handed B-DNA double helix. Z-DNA is left-handed form of the helix in which all bases are in anti-conformation. The phosphates in the backbone follow a zigzag path; hence, it is called as Z-DNA^24^. Compared to B-DNA, Z-DNA is not favoured thermodynamically. Although Z-DNA was shown to be formed in purine-pyrimidine repeats (d(CG)n) as a result of decreased electrostatic repulsion under high salt concentrations^25^, more recently, anion species were also shown to play a critical role in the formation and stabilization of Z-DNA^26^. Moreover, negative supercoiling and chemical base modifications were claimed to stabilize Z-DNA^27–29^. To determine whether the binding of DMSO to Z-form of DNA versus B-form of DNA was energetically more favourable, docking reactions were carried using AutoDock. 30 docking files were obtained for each case, and the positions with the lowest energy are shown in Fig. 5. In both Z-DNA and B-DNA, DMSO interacted with the amine (NH_2_) group of guanine base via hydrogen bond between DMSO’s oxygen moiety and one of the hydrogens of NH_2_ (Fig. 5A). Another important detail is that DMSO was bound to the minor groove of B-DNA (Fig. 5B), probably due to the orientation of guanine’s amine group always facing the minor groove. Binding energy for DMSO docked to Z-DNA was −2.57 Kcal/mol, while that for B-DNA was −2.48 Kcal/mol, giving a difference of −0.09 Kcal/mol, which in physiological conditions would require an increase in temperature of 45 °C to occur spontaneously. This indicates that DMSO binds to Z-DNA more strongly than to B-DNA.

When the space occupied by DMSO in Z-DNA was analysed in detail, it was found that Z-DNA could hold a second DMSO molecule parallel to the first. As shown in Fig. 5C, the two sides of the structure were perfect mirror images of each-other. However, it was not the same for the B-DNA conformation as two molecules could not be fitted in the structure. To determine whether the binding of two DMSO molecules to the DNA was energetically favourable, a mirror image of the docked DMSO on the complementary strand of Z-DNA was created and energy calculations were carried out. Since AutoDock was unable to dock more than one DMSO molecule to the DNA at a time, DFT calculations for the relative energies of none, one, and two-DMSO bound Z-DNA structures were carried out using B3LYP/6–31 G(d) in Gaussian. The results showed that binding of a single DMSO to a Z-DNA stabilizes the system by ca. 50 Kcal/mol, and binding of two DMSO molecules to the same Z-DNA stabilizes the system by ca. 90 Kcal/mol compared to the energy of naked Z-DNA (Z-DNA with two non-interacting DMSO positioned far away). These energy differences are remarkably high and cannot be overcome at the normal physiological temperature. ATR-FTIR data indicated an increase in the enhanced Z-form of DNA in the presence of DMSO (Fig. 4); we think that this increase could have resulted from the DMSO induced stabilization of Z-DNA. The results demonstrate that Z-DNA bound to two DMSO molecules was the most stable structure, followed by Z-DNA bound to one DMSO and finally naked Z-DNA, which is known to be very unstable in physiologic conditions^30^.

Z-DNA formation in cells rely on sequence composition together with chromatin structure^31^. Besides, a variety of environmental factors such as a high degree of hydration, high ionic strengths, transition-metal complexes and spermine are known to stabilize the Z-form^32^. Additionally, negative supercoiling, Z-DNA binding proteins, bromination and cytosine methylation are among the biological factors to stabilize Z-DNA in cells^33–35^. DMSO was shown to destabilize DNA double helix structure at low solvent concentrations by forming hydrogen bonds with nucleic acids^36^. This property is exploited to enhance PCR amplification reactions by inhibiting secondary structures in the DNA template or primers, especially during the synthesis of GC-rich gene fragments^37^. Prior studies showed that DMSO can denature duplex DNA because of its hydrophobic character^38^. While DMSO concentrations used in this study are not adequate to disrupt the duplex structure^38,39^, it may promote local conformational changes in DNA, as suggested by Kashino *et al*.^40^. By forming hydrogen bonds with DNA bases, DMSO may potentially help alter the helix conformation towards Z-form, in agreement with very early findings, which suggest that some regions of covalently-closed-circular DNA become more loose or more negatively supercoiled by the addition of 5.0% (v/v) DMSO^39^. Therefore, initiation and elongation of RNA synthesis may be enhanced at some locally relaxed regions of DNA^41,42^. In fact, there is a strong correlation between Z-DNA formation and active transcription^31^. There are several reports demonstrating that even at very low concentrations (0.05–2.5%, v/v), DMSO can affect gene expression^43–50^ and induces differentiation of many cell types including leukemia cells, mesenchymal stem cells, embryonic carcinoma cells, and preosteoblasts^5,51–53^. DMSO is also known to epigenetically alter DNA methylation profile^54–56^. Addition of DMSO (0.02–1.0%, v/v) to mouse embryoid bodies (EBs) affected genome-wide DNA methylation status, by changing hypermethylation as well as hypomethylation of genes and intergenic regions in EBs. Methylation-induced Z-DNA formation and stabilization is a known phenomenon^23,57–59^. Therefore, in the current study we suggest that gene expression changes and epigenetic modulations attributed to DMSO may be, at least in part, explained by the formation of Z-DNA. This in turn may affect numerous biological processes such as transcription regulation, nucleosome positioning, genetic instability, and chromatin remodelling^6,57,60^.

In summary, our experimental results, supported by *in silico* analyses unambiguously demonstrate that DMSO treatment significantly affected DNA conformation. To the best of our knowledge, we have described here for the first time that low concentrations of DMSO can change DNA helix topology to the Z-form. Although further advanced structural and biological studies are needed to understand the dynamic process of this phenomenon, this important finding emphasizes how the use of DMSO as a solvent may influence experimental results by interfering with gene expression.

### Low DMSO concentration can influence protein secondary structure

**Figure 6:**
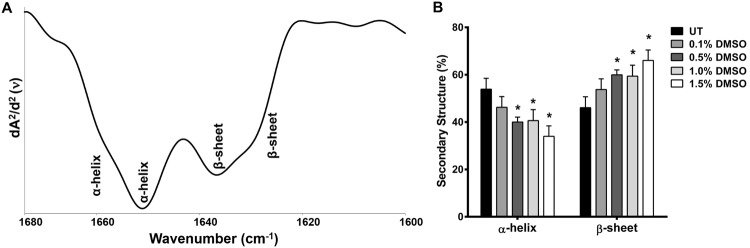
DMSO treatment affects protein secondary structures in HCT-116 cells. (**A**) The representative second derivative and vector-normalized IR spectra of HCT-116 cells in Amide I (1680–1600 cm^−1^) spectral region demonstrating protein secondary structure bands. (**B**) The band intensities (%) of total α-helix and β-sheet structures in DMSO treated and untreated (UT) HCT-116 cells.

To determine DMSO induced changes in the secondary structures of proteins, the second-derivative IR spectra of main secondary structural components in Amide I region (1680–1600 cm^−1^), considered as main protein region^61–63^, were examined. The two main α-helical structures located at 1657 and 1649 cm^−1^ and the two main β-sheet structures located at 1636 and 1629 cm^−1^ ^11,64^ are shown in Fig. 6A.

Treatment of HCT-116 cells with DMSO resulted in a dose dependent change in the conformation of proteins from α-helical to a β-sheet state starting at doses as low as 0.1% (v/v). We found that 0.1% DMSO treatment converted nearly 15% of α-helices to β-sheet conformation while treatment with 0.5% and 1.5% (v/v) resulted in conversion rates of 30% and 40%, respectively (Fig. 6B). Similar results were observed for SW-480 cells (Supplementary Fig. S8). Protein aggregates generally exhibit enhanced β-sheet content and diminished α-helicity compared to the native state, probably due to intermolecular contacts^65^. The formation of intermolecular β-sheets appears to be quite extensive in DMSO treated cells^66^. This calls for caution and awareness and the necessity of ensuring the physiochemical integrity as well as the biological activity of the analysed protein, since DMSO has the potential to cause protein denaturation, aggregation, or degradation, and even change the binding properties of the proteins^67^.

To the extent of our knowledge, this is the first study in which the impact of low doses of DMSO treatment on cellular proteins has been demonstrated. A large body of studies has consistently reported that DMSO can influence the properties of proteins in various ways. However, most of these studies were performed at very high DMSO concentrations (10–100%, v/v) with purified proteins^66,68–70^. Tjernberg *et al*. showed the effects of lower amounts of DMSO (0.1–3%, v/v) on two protein systems. These authors reported that DMSO could change the biochemical and binding properties of proteins in solution^67^. Functional and structural stability of proteins require a fine balance between the favourable internal interactions among residues of the protein and with solvent molecules^68^. It is suggested that DMSO destabilizes protein structure by perturbing the structure of water around the protein molecule which leads to DMSO induced protein unfolding^66,69,71^.

### DMSO affects membrane lipids

**Figure 7:**
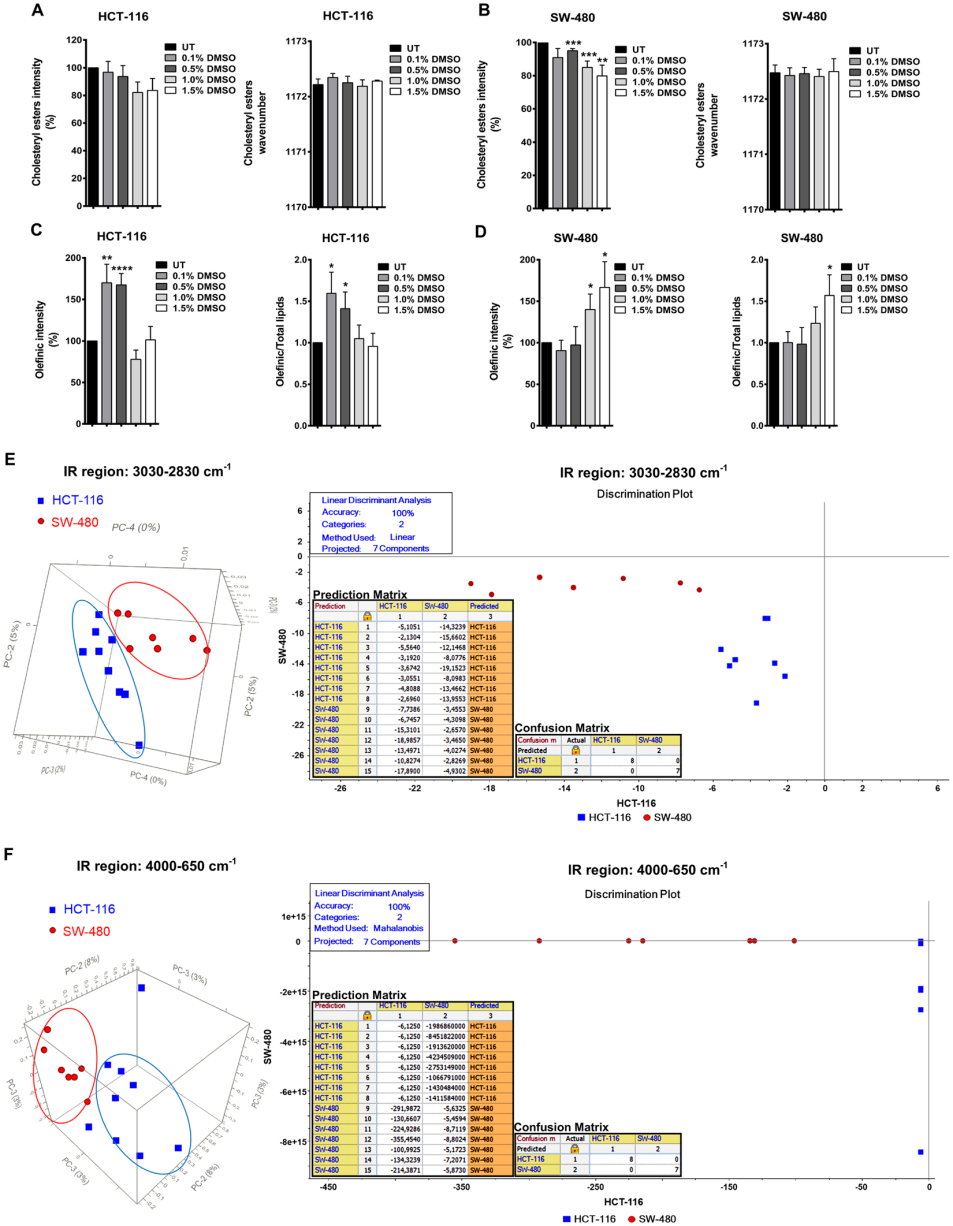
DMSO treatment influences cellular lipid content and status in HCT-116 and SW-480 cells. (**A**) The band intensities (%) and wavenumber values of cholesteryl esters in DMSO treated and untreated (UT) HCT-116 and, (**B**) in SW-480 cells. (**C**) The intensities (%) of olefinic and olefinic/total lipids bands in DMSO treated and UT HCT-116 and, (**D**) SW-480 cells. The results are represented as mean ± SEM. t test was used to compare the results with respect to untreated cells. (**E**) Left Panel: PCA 3D scores plots for HCT-116 and SW-480 cells in 3030–2830 cm^−1^ and 4000–650 cm^−1^ spectral regions. Right Panel: LDA discrimination plots, and prediction and confusion matrices for HCT-116 and SW-480 cells in 3030–2830 cm^−1^ and 4000–650 cm^−1^ spectral regions.

Quantitative and qualitative information on the effect of DMSO on membrane cholesterol in both HCT-116 and SW-480 cells was retrieved by analysing the band located at 1172 cm^−1^ in the fingerprint region of IR spectra assigned to CO–O–C antisymmetric stretching of the ester bonds in cholesteryl esters^13^. The intensity (%) of this band was dose dependently reduced in DMSO treated cells compared to the untreated control (UT) cells. However, we did not observe any differences in wavenumber values between untreated and DMSO treated samples indicating that DMSO does not alter molecular structure of cholesterol (Fig. 7A,B).

The thickness of membranes is related to their cholesterol and cholesteryl esters content, with thinner membranes containing less cholesterol^72^. Decreased membrane thickness with increased doses of DMSO may enhance membrane permeability and facilitate the transport of active molecules^73^. Thus, membrane associated effects attributed to DMSO, like enhancement of permeability, facilitation of membrane fusion, and enabling the cell membrane to accommodate osmotic and mechanical stresses during cryopreservation^74^, may be related to its ability to change the lipid composition and dynamics of biological membranes.

The reaction of free radicals with the olefinic bonds of polyunsaturated lipids results in lipid peroxidation, which in turn causes the loss of unsaturation in the lipid structure together with the breakdown of longer chains into smaller lipid acyl chains^14^. The olefinic band located at 3009 cm^−1^ in the lipid region of IR spectra assigned to unsaturated lipids was analysed to measure the level of unsaturation and lipid peroxidation end-products as a result of DMSO treatment. An increase in intensity of this unsaturation marker band^13^ is related to elevated concentrations of unsaturated acyl chains of lipids and loss of lipid-peroxidation end-products^75–77^. Specifically, intensities (%) of olefinic band and the ratio of olefinic to total lipids (olefinic/total lipid) were analysed to monitor the acyl chain unsaturation level of the phospholipids^14^ (Fig. 7C,D). In this, the sum of the intensities (%) of olefinic (3009 cm^−1^), CH_2_ antisymmetric (2921 cm^−1^), CH_2_ symmetric (2852 cm^−1^) and cholesteryl ester (1172 cm^−1^) bands were calculated as total lipids. The intensities (%) of olefinic band and olefinic/total lipid ratios (%) increased statistically significantly in 0.1% and 0.5% DMSO treated HCT-116 cells; however, higher DMSO doses reverted the effect (Fig. 7C). This may be caused by a negative feedback mechanism to maintain membrane phospholipid composition^78,79^. On the other hand, the intensities (%) of olefinic band and olefinic/total lipid ratios (%) were significantly increased in 1.0% and 1.5% DMSO treated SW-480 cells (Fig. 7D). It is possible that a similar reversion may occur in SW-480 at even higher doses of DMSO. Of note, the olefinic intensity (%) was similar to the olefininc/total lipid ratio, indicating that rather than inducing any alteration in lipid biosynthesis or degradation, the effect was on the peroxidation of existing lipid molecules. Thus, while low doses of DMSO were quite enough to elevate the unsaturation level and decrease lipid peroxidation end-products in HCT-116, higher doses of DMSO were needed for this purpose in SW-480 cells.

The relative susceptibility of individual fatty acids to peroxidation depends on the membrane fatty acid composition^80^. Tapiero *et al*. reported that the effects of DMSO on membranes were related to the structure and/or composition of the cell membranes that are different in various cell types^81^. Therefore, the different oxidative effects of DMSO in HCT-116 and SW-480 cells may be related to distinct membrane lipid characteristics of these cells. To demonstrate these differences, we ran unsupervised PCA and supervised LDA to compare HCT-116 and SW-480 cells, in lipid (3030–2830 cm^−1^) (Fig. 7E) and whole (4000–650 cm^−1^) IR regions (Fig. 7F). The PCA results are given in the left and the LDA results are presented in the right panel of Fig. 7. PCA 3D scores plots (Fig. 7E,F; left panel) revealed a clear-cut segregation between HCT-116 and SW-480 cells, confirming our hypothesis of inherent differences between these cell lines. LDA discrimination plots, and prediction and confusion matrices (Fig. 7E,F; right panel) similarly revealed a well-defined discrimination between HCT-116 and SW-480 cells with 100% accuracy. Moreover, the obvious spectral differences between these two cell types were prominent in the lipid (4000–2800 cm^−1^) and fingerprint (1800–650 cm^−1^) IR spectra as demonstrated in Supplementary Fig. S9A,B, respectively. To further examine whether discrimination between SW-480 and HCT-116 cells with such high accuracy was due to differential gene expression related to lipid metabolism, we examined a publicly available microarray dataset (GSE41445) that reported gene expression of wild type HCT-116 and SW-480 cells. We observed that nearly double the number of genes (61 genes in SW-480 cells versus 37 genes in HCT-116 cells) related to the gene ontology (GO) term LIPID_METABOLISM were statistically significantly (p < 0.05) differentially expressed with fold change of 1 between the two cell lines (Supplementary Fig. S10). Thus the effects of DMSO on the lipid component of the two cell lines examined may have resulted from genotypic differences between the cell lines.

## Conclusion

DMSO is a widely used organic solvent for biological applications because of its capability to dissolve numerous polar or nonpolar compounds. In high throughput screening assays for drug discovery, most small molecule compounds, including potential anti-cancer agents, are dissolved in DMSO^82,83^. For the first time, in the present study the effects of low, non-toxic doses of DMSO on colon cancer epithelial cells, at concentrations that are widely used in biological research, were investigated in detail by Infrared (IR) spectroscopy coupled with pattern recognition techniques and confirmed by molecular biology techniques and *in silico* analyses. We observed that treatment of two different colon epithelial cell lines with DMSO increased β-sheet formation in proteins, decreased cholesteryl esters, and affected cellular lipid content and oxidation status. In addition, here we show that DMSO treatment reduced the total nucleic acid content and affected DNA topology by enhancing Z-form of DNA substantially in both tumorigenic and non-tumorigenic epithelial cells. In the presence of DMSO, stabilization of Z-DNA over B-DNA was further supported by molecular docking and free energy calculation studies. The merit of understanding the effects of DMSO on Z-DNA formation/stabilization is that it will help in clarifying the role of Z-DNA in a biological context.

**Figure 8:**
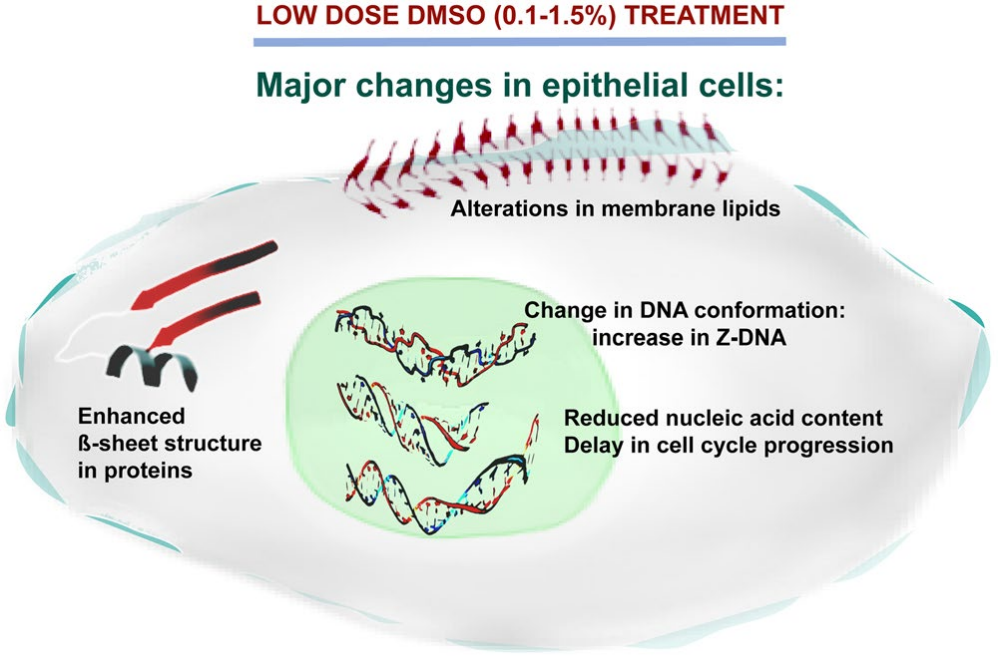
Overall model showing the effects of low dose (0.1–1.5%) DMSO on epithelial cells. In epithelial phenotype cells, DMSO can induce significant macromolecular changes that include alterations in membrane phospholipid composition and/or oxidation status of existing lipid molecules, decrease in the nucleic acid content, delayed cell cycle progression, perturbations of protein secondary structure and substantially enhanced Z-form of DNA.

The observed changes in gross macromolecular structures (Fig. 8) can address many of the applications of DMSO in cell biology as well highlight some of the pitfalls of using DMSO as a solvent. Thus, it is the hope of the authors that this report will bring researcher’s attention to exhibit caution when interpreting data and conclusions in experiments where DMSO is used as a solvent, since even low concentrations of DMSO may interfere with important cellular processes as demonstrated in this study.

## Methods

### Cell culture and treatments

HCT-116 cells were purchased from Deutsche Sammlung von Mikroorganismen und Zellkulturen (DSMZ, Germany), and SW-480 cells were obtained from ATCC (England). RMPI-1640 and DMEM supplemented with 10% Fetal Bovine Serum (FBS), 2 mM L-glutamine and 1% penicillin/streptomycin were used to grow HCT-116 and SW-480 cells, respectively. MCF-10A cells, kindly provided by Dr. Elif Erson Bensan (Orta Dogu Teknik Universitesi, Ankara, Turkey), were grown in DMEM/F12 medium supplemented with 5% horse serum, 20 ng/ml epidermal growth factor (EGF), 0.5 mg/ml hydrocortisone, 100 ng/ml cholera toxin and 10 µg/ml insulin and 1% penicillin/streptomycin. Cells were cultured in a humidified atmosphere containing 5% CO_2_ at 37 °C. All cell culture consumables were purchased from Biological Industries (Beit Haemek, Israel). Cells were either left untreated or treated with 0.1%, 0.5%, 1.0% and 1.5% cell culture grade DMSO (PanReac AppliChem, Germany) for indicated times.

### IR spectroscopy measurements and data analyses

The cell culture medium was aspirated and the wells washed with cell culture grade Phosphate-buffered saline (PBS). Cells were then detached using Trypsin-EDTA (0.25%) solution. The trypsin was neutralized by adding fresh medium. The cells were centrifuged, the pellet was washed in PBS, followed by centrifugation at 400 × *g* for 7 min, to remove the PBS. The cells were resuspended in PBS, counted with a haemocytometer and adjusted to 5 × 10^5^ cells/10 µL of PBS for ATR-FTIR.

The IR spectra of untreated and DMSO treated cells were obtained using Spectrum 100 FTIR spectrometer (PerkinElmer, Waltham, Massachusetts, USA) equipped with a Universal ATR accessory. The spectrum of air was used as a reference. 10 µL of sample, corresponding to 5 × 10^5^ cells in PBS, was placed on a diamond/ZnSe crystal plate (PerkinElmer) and dried with a mild nitrogen flux for 2 min. Purging samples with non-invasive N_2_ was applied in order to remove the overlapping free water bands from the samples (while keeping the bound water in the system), a common procedure in ATR-FTIR studies^84^. PBS, which was used during the sample preparation step, was scanned under identical experimental conditions as the samples. These spectra were manually subtracted from the sample spectra using the Spectrum 100 software (PerkinElmer, Waltham, Massachusetts, USA). During the subtraction process, the free water band located around 2125 cm^−1^ was flattened using the same software in order to eliminate the effect of PBS. The difference spectra obtained were used for all further spectral analyses^11^. For HCT-116 and SW-480 cells, three biological replicates each with three technical replicates, and for MCF-10A cells, two biological replicates each with four technical replicates were analysed. The samples were scanned over the spectral range 4000 to 650 cm^−1^, at room temperature, with a resolution of 4 cm^−1^ and spectra were collected as an average of 64 scans.

Pre-processing and/or standardization of FT-IR data are essential for accurate interpretation. Vector normalization and mean-centering by using second derivative spectra in the full and/or restricted fingerprint regions (ideal for quantitative analyses) is commonly used and is considered to be a reliable method for pre-processing of FT-IR data. In vector normalization, each spectrum is divided by the Euclidean norm and is usually applied to second derivative spectra because they contain both positive and negative values (below zero). Min/max normalization is another method in which the standardization is carried out on a single and stable peak (Amide I and II). Compared with vector normalization, min/max normalization method is often used for visual and qualitative purposes, since it can only process spectra with positive values^85^.

During data analyses, the second derivative and vector-normalized IR spectra were used in order to increase the accuracy of band positions. However, for the determination of absolute intensities of Amide bands (proteins) and PO_2_ antisymmetric and symmetric bands (total nucleic acids), vector-normalized absorbance IR spectra were used. Opus 5.5 software (Bruker, Billerica, Massachusetts, USA) was utilized for all data pre-processing. IR analysis of the absolute band intensities of untreated groups were normalized to 100%, and DMSO treated groups were calculated accordingly and expressed as percent intensity.

### Principal component analysis (PCA)

PCA is an unsupervised modelling technique that transforms a group of equivalent variables into a smaller set of distinct variables called principal components (PCs), while preserving the vast majority of actual information. Thus, a reduced size PC model can be employed to rapidly identify and differentiate abnormalities/deviations in the original system^86^. There are different criteria for selecting the number PCs, since there is no universally accepted and unconditional methodology^87^. Higher PCs (PC1 and PC2) describe the predominant sources of variations in the dataset and generally explain the gross spectral changes that may mask smaller ones^20,88^. This strategy is preferable when performing robust analysis of the bulk data. Another strategy is the analysis of subsequent minor PCs that should be considered for the detection of much smaller but more distinctive and itemized alterations occurring in macromolecules^89^.

PCA was applied to ATR-FTIR spectral data in order to perform an exploratory analysis of the studied experimental groups. For this method, unit vector-normalized and mean-centered spectra of each group were imported into *The Unscrambler® X 10*.*3* (CAMO Software AS, Norway) multivariate analysis (MVA) software. PCA was applied in the 4000–650 cm^−1^, 3030–2830 cm^−1^ and 1250–1200 cm^−1^ IR regions using Nonlinear Iterative Partial Least Squares (NIPALS) algorithm and the results were presented as three-dimensional (3D) scores plots. Hotelling’s T2 statistics were used in scores plots to monitor major variations within the PCA model.

### Linear discriminant analysis (LDA)

LDA is a supervised modelling technique that involves linearly transforming n-dimensional feature samples into an m-dimensional space (m < n). In this way, members of the same class can be categorized together while members of different classes cluster separately, maximizing the differences between the predefined classes, with respect to the new variable^90^.

LDA was performed using *The Unscrambler*^*®*^ *X 10*.*3* (CAMO Software AS, Norway) multivariate analysis (MVA) software. The modelling was based on PC scores. The category variable column was included into a data matrix and subsequently, randomly chosen spectra among the different sample categories was used to build a training set. After defining the data for modelling, Mahalanobis and/or Linear classifiers methods were used for LDA. The results are presented as discrimination plots, as well as prediction and confusion matrices.

### Western blot analysis

Cells were lysed in M-PER Mammalian Protein Extraction Reagent (Thermo Fisher Scientific, Waltham, Massachusetts, USA) containing protease inhibitor cocktail (Roche, Switzerland) and phosphatase inhibitor (Roche, Switzerland) according to manufacturer’s instructions. Proteins (20–50 µg) were separated in 10% SDS-PAGE gels at 100 V and transferred to polyvinylidene difluoride (PVDF) membranes using standard techniques. After 1 h of blocking in 5% skim-milk in TBS-T (Tris-buffered saline, 0.1% Tween 20), the membranes were incubated in primary antibodies (Supplementary Table S1) overnight at 4 °C, followed by 1 h incubation with HRP-conjugated secondary antibody at room temperature. Bands were visualized by using Clarity ECL Substrate (Bio-Rad, Hercules, California, USA) in a ChemiDoc MP Imaging System (Bio-Rad).

### MTT assay

To evaluate the effect of DMSO on cell viability, Vybrant^®^ MTT (3-(4,5-dimethylthiazol-2-yl)-2,5-diphenyltetrazolium bromide) Cell Proliferation Assay (Thermo Fisher Scientific) was used according to the manufacturer’s instructions. Briefly, cells were seeded as 5 × 10^3^ cells/well of a 96-well plate and allowed to attach overnight. The day after, the cells were incubated with different doses of DMSO (0.1–1.5%) or left untreated. After 24 h of incubation with DMSO, the medium was replaced with 100 µL of complete culture medium containing 1.2 mM of MTT. Following 4 h of incubation at 37 °C, 100 µL of the SDS-HCl solution (1 g of SDS in 10 mL of 0.01 M HCl) was added to each well and mixed thoroughly by pipetting. The plates were incubated at 37 °C for 16 h in a humidified atmosphere at 37 °C. The absorbances were measured at 570 nm in a microplate reader (Thermo Fisher Scientific), and relative cell viability (%) was expressed as a percentage relative to the untreated control cells.

### Cell cycle and DNA content analysis

To determine the effect of DMSO on cell cycle distribution, cells were treated with indicated DMSO doses for indicated times. The cells were synchronized at the G1 phase by incubating the cells in serum free medium. After 24 h, the serum free medium was removed, and the cells were incubated with complete growth medium containing 10% FBS for 7 h to release from G1.

For cell cycle analysis, DMSO treated and untreated cells were detached in trypsin/EDTA, and washed once in ice cold PBS (Phosphate-buffered saline), before fixation in 70% cold ethanol. The fixed cells were washed with PBS two times, resuspended in a staining solution consisting of 0.1% Triton X-100, 20 μg/mL RNase A (Thermo Fisher Scientific) and 20 µg/mL Propidium Iodide (Sigma-Aldrich Chemie GmbH, Germany) in PBS and incubated for 30 min in the dark at room temperature. The percentage of cells at different stages of cell cycle was determined by using Accuri C6 Flow Cytometer (BD Biosciences, Franklin Lakes, New Jersey, USA) in the FL-3 channel.

### Dihydroethidium (DHE) assay

To measure intracellular ROS levels in DMSO treated and untreated cells, HCT-116 and SW-480 cells were incubated with indicated doses of DMSO for 24 or 48 h, detached by trypsinization and collected by centrifugation at 400 × *g* for 7 min. After washing in PBS, cells were incubated in DHE (Thermo Fisher Scientific) staining solution (3.2 μM in PBS) for 20 min at 37 °C, and immediately analysed by Accuri C6 Flow Cytometer (BD Biosciences) in the FL-2 channel.

### Auto dock

In order to assess the interactions between DNA and DMSO, molecular docking analyses were carried out with AutoDock4.2 software^91^. To determine whether the binding affinities of DMSO to Z-DNA versus B-DNA were different, high resolution (1.6 Å for B-DNA and 1.0 Å for Z-DNA) X-ray structures containing a complete helical turn with the sequences TACGCCCACGC (11-mer) for B-DNA (PDB ID 1AAY)^92^ and CGCGCG (6-mer) for Z-DNA (PDB ID 1DCG)^30^ were chosen from the PDB repository. The coordinates of DMSO were downloaded in.sdf format from PubChem Public database (PubChem CID 679) and converted into pdbqt format by OpenBabel2.4.1 software^93^. Docking was performed as described previously^94,95^. Briefly, protein components, ions and water were removed from the PDB DNA structures. The chosen parameters were as follows: in the gridding step, the entire sequence (6 mer, CGCGCG) of the Z-DNA was included whereas for the B-DNA, only the GC-rich region (CGCCC) was included. The choice of sequence of B-DNA was based on preliminary optimizations by starting with the whole molecule and then narrowing down to this region for better comparison with the Z-DNA sequence. For docking, the Genetic Algorithm was used for searching with 30 runs and Lamarkian GA to write the output.dpf file. The parameter chosen for best position was “Binding Energy” and the values calculated from empirical free energy scoring function for all 30 structures are shown in Supplementary Table S2. The images were generated with UCSF Chimera software version 1.11.2^96^.

### Gaussian

The most stable Z-DNA-DMSO complex, determined from molecular docking analysis, was used for further structural analysis to determine the stability of two DMSO molecules docked in the Z-DNA structure relative to one molecule of DMSO and none. DFT computational results were obtained by using B3LYP/6–31 G(d) functional/basis set combination in Gaussian 09, Revision A.02 software package^97^ at TÜBİTAK ULAKBİM, High Performance and Grid Computing Centre (TRUBA) and visualization of the output files was carried out using Chemcraft software (https://www.chemcraftprog.com). The initial structure of Z-DNA with two DMSO molecules was obtained from a stable Z-DNA-DMSO complex by adding a second DMSO molecule to the opposite strand mirroring the coordinates of the first. Two and one extra DMSO for none and one-DMSO cases, respectively, were added to the calculations and positioned far away from the Z-DNA in order to ensure no interaction between the Z-DNA and DMSO. That way equal number of atoms was achieved during the calculations for the three cases in order to make the resulted energies comparable. Prior to energy calculations the structures were optimized with PM3. During the optimization, the Z-DNA structure was frozen while DMSO molecules were left free to move to obtain energetically the most stable structure.

### Bioinformatic data analysis

Raw data for GSE41445 was downloaded using GEOquery package from *bioconductor*^98^. The data was normalized using *gcrma*^99^ and annotated using hgu133plus2.db^100^ packages, respectively. Differential gene expression analysis for HCT-116 and SW-480 were performed using *limma*^101^ package and the genes related to lipid metabolism (GO:0006629) were extracted using *stringr*^102^ package based on the Affymetrix annotation file. All analyses were performed on R Language Software version 3.5.1^103^.

### Statistical data analysis

The results were expressed as mean ± standard error of mean (SEM). t test was used for comparisons using Prism 6.01 (GraphPad, La Jolla, California, USA). The degree of significance was denoted as *p ≤ 0.05, **p ≤ 0.01, ***p ≤ 0.001, ****p ≤ 0.0001.

## Electronic supplementary material

Supplementary Figures S1 - S11

## Data Availability

All data generated during and/or analysed during the current study are available from the corresponding author on reasonable request. The full length western blot images are shown in Supplementary Fig. S11.

## References

[1] Capriotti K, Capriotti JA (2012). Dimethyl sulfoxide: History, chemistry, and clinical utility in dermatology. J. Clin. Aesthet. Dermatol..

[2] Sanmartín-Suárez C, Soto-Otero R, Sánchez-Sellero I, Méndez-Álvarez E (2011). Antioxidant properties of dimethyl sulfoxide and its viability as a solvent in the evaluation of neuroprotective antioxidants. J. Pharmacol. Toxicol. Methods.

[3] Hall MD (2014). Say no to DMSO: Dimethylsulfoxide inactivates cisplatin, carboplatin, and other platinum complexes. Cancer Res..

[4] Fiore M, Zanier R, Degrassi F (2002). Reversible G(1) arrest by dimethyl sulfoxide as a new method to synchronize Chinese hamster cells. Mutagenesis.

[5] Santos NC, Figueira-Coelho J, Martins-Silva J, Saldanha C (2003). Multidisciplinary utilization of dimethyl sulfoxide: Pharmacological, cellular, and molecular aspects. Biochemical Pharmacology.

[6] Jacob, S. W. & Torre, J. C. de la. *Dimethyl Sulfoxide (DMSO) in Trauma and Disease* CRC Press (2015).

[7] Michalski R, Michalowski B, Sikora A, Zielonka J, Kalyanaraman B (2014). On the use of fluorescence lifetime imaging and dihydroethidium to detect superoxide in intact animals and *ex vivo* tissues: A reassessment. Free Radic. Biol. Med..

[8] Zhao H (2003). Superoxide reacts with hydroethidine but forms a fluorescent product that is distinctly different from ethidium: Potential implications in intracellular fluorescence detection of superoxide. Free Radic. Biol. Med..

[9] Wang L, Mizaikoff B (2008). Application of multivariate data-analysis techniques to biomedical diagnostics based on mid-infrared spectroscopy. Anal. Bioanal. Chem..

[10] Stuart, B. H. *Infrared Spectroscopy: Fundamentals and Applications*. (John Wiley and Sons Ltd, 2004).

[11] Gurbanov R, Bilgin M, Severcan F (2016). Restoring effect of selenium on the molecular content, structure and fluidity of diabetic rat kidney brush border cell membrane. Biochim. Biophys. Acta - Biomembr..

[12] Banyay M, Sarkar M, Gräslund A (2003). A library of IR bands of nucleic acids in solution. Biophysical Chemistry.

[13] Ozek NS, Tuna S, Erson-Bensan aE, Severcan F (2010). Characterization of microRNA-125b expression in MCF7 breast cancer cells by ATR-FTIR spectroscopy. Analyst.

[14] Inan Genç A, Gok S, Banerjee S, Severcan F (2017). Valdecoxib Recovers the Lipid Composition, Order and Dynamics in Colon Cancer Cell Lines Independent of COX-2 Expression: An ATR-FTIR Spectroscopy Study. Appl. Spectrosc..

[15] William R (1997). Granulocytic differentiation of HL-60 cells results in spontaneous apoptosis mediated by increased caspase expression. FEBS Lett..

[16] Sawai M, Takase K, Teraoka H, Tsukada K (1990). Reversible G1 arrest in the cell cycle of human lymphoid cell lines by dimethyl sulfoxide. Exp. Cell Res..

[17] Teraoka H (1991). Expression of c-fos and c-myc in Raji Burkitt’s lymphoma cells during the progression of DMSO-induced G1 cells into S phase. Exp. Cell Res..

[18] Chetty S (2013). A simple tool to improve pluripotent stem cell differentiation. Nat. Methods.

[19] Burhans WC, Heintz NH (2009). The cell cycle is a redox cycle: Linking phase-specific targets to cell fate. Free Radical Biology and Medicine.

[20] Whelan DR (2011). Monitoring the reversible B to A-like transition of DNA in eukaryotic cells using Fourier transform infrared spectroscopy. Nucleic Acids Res..

[21] Zhang F, Huang Q, Yan J, Chen Z (2016). Histone Acetylation Induced Transformation of B-DNA to Z-DNA in Cells Probed through FT-IR Spectroscopy. Anal. Chem..

[22] Severcan F, Haris PI (2012). Introduction to vibrational spectroscopy in diagnosis and screening. Adv. Biomed. Spectrosc..

[23] Dumat Blaise, Larsen Anders Foller, Wilhelmsson L. Marcus (2016). Studying Z-DNA and B- to Z-DNA transitions using a cytosine analogue FRET-pair. Nucleic Acids Research.

[24] Herbert A, Rich A (1999). Left-Handed Z-Dna: Structure and Function. Genetica.

[25] Doluca O, Withers JM, Filichev VV (2013). Molecular engineering of guanine-rich sequences: Z-DNA, DNA triplexes, and G-quadruplexes. Chemical Reviews.

[26] Bae S, Son H, Kim Y-G, Hohng S (2013). Z-DNA stabilization is dominated by the Hofmeister effect. Phys. Chem. Chem. Phys..

[27] Peck LJ, Nordheim A, Rich A, Wang JC (1982). Flipping of cloned d(pCpG)n.d(pCpG)n DNA sequences from right- to left-handed helical structure by salt, Co(III), or negative supercoiling. Proc Natl Acad Sci USA.

[28] Haniford DB, Pulleyblank DE (1983). The *in-vivo* occurrence of Z DNA. J. Biomol. Struct. Dyn..

[29] Behe M, Felsenfeld G (1981). Effects of methylation on a synthetic polynucleotide: the B–Z transition in poly(dG-m5dC).poly(dG-m5dC). Proc. Natl. Acad. Sci. USA.

[30] Gessner RV, Frederick CA, Quigley GJ, Rich A, Wang AHJ (1989). The molecular structure of the left-handed Z-DNA double helix at 1.0-Å atomic resolution. Geometry, conformation, and ionic interactions of d(CGCGCG). J. Biol. Chem..

[31] Shin SI (2016). Z-DNA-forming sites identified by ChIP-Seq are associated with actively transcribed regions in the human genome. DNA Res..

[32] Eun, H-M. Enzymes and Nucleic Acids: General Principles in *Enzymology Primer for Recombinant DNA Technology* 1–108 (Academic Press, 1996).

[33] Rich A, Nordheim A, Azorin F (1983). Stabilization and detection of natural left-handed z-dna. J. Biomol. Struct. Dyn..

[34] Neidle Stephen (2008). DNA Structure as Observed in Fibers and Crystals. Principles of Nucleic Acid Structure.

[35] Rich A, Zhang S (2003). Timeline: Z-DNA: the long road to biological function. Nat. Rev. Genet..

[36] Nakano S, Sugimoto N (2016). The structural stability and catalytic activity of DNA and RNA oligonucleotides in the presence of organic solvents. Biophys. Rev..

[37] Winship PR (1989). An improved method for directly sequencing PCR amplified material using dimethyl sulphoxide. Nucleic Acids Res..

[38] Lee J, Vogt CE, McBrairty M, Al-Hashimi HM (2013). Influence of dimethylsulfoxide on RNA structure and ligand binding. Anal. Chem..

[39] Wang X, Lim HJ, Son A (2014). Characterization of denaturation and renaturation of DNA for DNA hybridization. Environ. Health Toxicol..

[40] Kashino G (2010). An alternative mechanism for radioprotection by dimethyl sulfoxide; possible facilitation of DNA double-strand break repair. J. Radiat. Res..

[41] Juang JK, Liu HJ (1987). The effect of DMSO on natural DNA conformation in enhancing transcription. Biochem. Biophys. Res. Commun..

[42] Wölfl S, Martinez C, Rich A, Majzoub JA (1996). Transcription of the human corticotropin-releasing hormone gene in NPLC cells is correlated with Z-DNA formation. Proc. Natl. Acad. Sci. USA.

[43] Adler S, Paparella M, Pellizzer C, Hartung T, Bremer S (2005). The detection of differentiation-inducing chemicals by using green fluorescent protein expression in genetically engineered teratocarcinoma cells. Altern Lab Anim.

[44] Klinken SP, Holmes KL, Morse HC, Thorgeirsson SS (1988). Transcriptional and post-transcriptional regulation of c-myc, c-myb, and p53 during Proliferation and differentiation of murine erythroleukernia cells treated with DFMO and DMSO. Exp. Cell Res..

[45] Jiang G (2006). Down-regulation of TRRAP-dependent hTERT and TRRAP-independent CAD activation by Myc/Max contributes to the differentiation of HL60 cells after exposure to DMSO. Int. Immunopharmacol..

[46] Nishimura M, Nikawa T, Kawano Y, Nakayama M, Ikeda M (2008). Effects of dimethyl sulfoxide and dexamethasone on mRNA expression of housekeeping genes in cultures of C2C12 myotubes. Biochem. Biophys. Res. Commun..

[47] Nishimura M, Ueda N, Naito S (2003). Effects of dimethyl sulfoxide on the gene induction of cytochrome P450 isoforms, UGT-dependent glucuronosyl transferase isoforms, and ABCB1 in primary culture of human hepatocytes. Biol. Pharm. Bull..

[48] Wilkening S, Bader A (2004). Differential regulation of CYP3A4 and CYP3A7 by dimethylsulfoxide in primary human hepatocytes. Basic Clin. Pharmacol. Toxicol..

[49] Choi S (2009). Characterization of increased drug metabolism activity in dimethyl sulfoxide (DMSO)-treated Huh7 hepatoma cells. Xenobiotica..

[50] Sumida K (2011). Effects of DMSO on gene expression in human and rat hepatocytes. Hum. Exp. Toxicol..

[51] Okura H (2010). Cardiomyoblast-like cells differentiated from human adipose tissue-derived mesenchymal stem cells improve left ventricular dysfunction and survival in a rat myocardial infarction model. Tissue Eng. Part C. Methods.

[52] Hegner B, Weber M, Dragun D, Schulze-Lohoff E (2005). Differential regulation of smooth muscle markers in human bone marrow-derived mesenchymal stem cells. J. Hypertens..

[53] Seya K (2012). Br-DIF-1 accelerates dimethyl sulphoxide-induced differentiation of P19CL6 embryonic carcinoma cells into cardiomyocytes. Br. J. Pharmacol..

[54] Iwatani M (2006). Dimethyl sulfoxide has an impact on epigenetic profile in mouse embryoid body. Stem Cells.

[55] Friend C, Scher W, Holland JG, Sato T (1971). Hemoglobin synthesis in murine virus-induced leukemic cells *in vitro*: stimulation of erythroid differentiation by dimethyl sulfoxide. Proc. Natl. Acad. Sci. USA.

[56] Bonser RW, Siegel MI, McConnell RT, Cuatrecasas P (1981). The appearance of phospholipase and cyclo-oxygenase activities in the human promyelocytic leukemia cell line HL60 during dimethyl sulfoxide-induced differentiation. Biochem. Biophys. Res. Commun..

[57] Zacharias W, Jaworski A, Wells RD (1990). Cytosine methylation enhances Z-DNA formation *in vivo*. J. Bacteriol..

[58] Kagawa K, Howell ML, Tseng K, Ho PS (1993). Effects of base substituents on the hydration of B- and ZDNA: Correlations to the B- to Z-DNA transition. Nucleic Acids Res..

[59] Temiz Nuri A., Donohue Duncan E., Bacolla Albino, Luke Brian T., Collins Jack R. (2012). The Role of Methylation in the Intrinsic Dynamics of B- and Z-DNA. PLoS ONE.

[60] Rich A, Zhang S (2003). Z-DNA: the long road to biological function. Nature.

[61] Toyran N, Lasch P, Naumann D, Turan B, Severcan F (2006). Early alterations in myocardia and vessels of the diabetic rat heart: an FTIR microspectroscopic study. Biochem. J..

[62] Barth A (2007). Infrared spectroscopy of proteins. Biochimica et Biophysica Acta - Bioenergetics.

[63] Bozkurt O, Severcan M, Severcan F (2010). Diabetes induces compositional, structural and functional alterations on rat skeletal soleus muscle revealed by FTIR spectroscopy: a comparative study with EDL muscle. Analyst.

[64] Yang H, Yang S, Kong J, Dong A, Yu S (2015). Obtaining information about protein secondary structures in aqueous solution using Fourier transform IR spectroscopy. Nat. Protoc..

[65] Mande SC, Sobhia ME (2000). Structural characterization of protein-denaturant interactions: crystal structures of hen egg-white lysozyme in complex with DMSO and guanidinium chloride. Protein Eng..

[66] Greve TM, Andersen KB, Nielsen OF (2008). Penetration mechanism of dimethyl sulfoxide in human and pig ear skin: An ATR-FTIR and near-FT Raman spectroscopic *in vivo* and *in vitro* study. Spectroscopy.

[67] Tjernberg A (2005). DMSO-Related Effects in Protein Characterization. J. Biomol. Screen..

[68] Batista ANL, Batista JM, Bolzani VS, Furlan M, Blanch EW (2013). Selective DMSO-induced conformational changes in proteins from Raman optical activity. Phys. Chem. Chem. Phys..

[69] Huang P, Dong A, Caughey WS (1995). Effects of dimethyl sulfoxide, glycerol, and ethylene glycol on secondary structures of cytochrome c and lysozyme as observed by infrared spectroscopy. J. Pharm. Sci..

[70] Roy Susmita, Jana Biman, Bagchi Biman (2012). Dimethyl sulfoxide induced structural transformations and non-monotonic concentration dependence of conformational fluctuation around active site of lysozyme. The Journal of Chemical Physics.

[71] Arakawa T, Kita Y, Timasheff SN (2007). Protein precipitation and denaturation by dimethyl sulfoxide. Biophys. Chem..

[72] Choi MK (2016). Maintenance of membrane integrity and permeability depends on a patched-related protein in Caenorhabditis elegans. Genetics.

[73] He F (2012). Ion transport through dimethyl sulfoxide (DMSO) induced transient water pores in cell membranes. Mol. Membr. Biol..

[74] Notman R, Noro M, Malley BO, Anwar J (2006). Molecular Basis for Dimethylsulfoxide (DMSO) Action on Lipid Membranes Molecular Basis for Dimethylsulfoxide (DMSO) Action on Lipid Membranes. J. Amer. Chem. Soc..

[75] Sills RH, Moore DJ, Mendelsohn R (1994). Erythrocyte peroxidation: quantitation by Fourier transform infrared spectroscopy. Anal. Biochem..

[76] Severcan F, Gorgulu G, Gorgulu ST, Guray T (2005). Rapid monitoring of diabetes-induced lipid peroxidation by Fourier transform infrared spectroscopy: Evidence from rat liver microsomal membranes. Anal. Biochem..

[77] Turker S, Ilbay G, Severcan M, Severcan F (2014). Investigation of compositional, structural, and dynamical changes of pentylenetetrazol-induced seizures on a rat brain by FT-IR spectroscopy. Anal. Chem..

[78] Pamplona R (2008). Membrane phospholipids, lipoxidative damage and molecular integrity: A causal role in aging and longevity. Biochimica et Biophysica Acta - Bioenergetics.

[79] Fernandez ML, West KL (2005). Mechanisms by which dietary fatty acids modulate plasma lipids. J. Nutr..

[80] Naudí, A. *et al*. Membrane lipid unsaturation as physiological adaptation to animal longevity. *Frontiers in Physiology* **4** (2013). 10.3389/fphys.2013.00372 PMC3865700 24381560

[81] Tapiero H, Zwingelstein G, Fourcade A, Portoukalian J (1983). The effect of dimethyl sulfoxide on the membrane dynamics and the phospholipid composition of two different cell lines. Ann N Y Acad Sci.

[82] Dandapani, S., Rosse, G., Southall, N., Salvino, J. M. & Thomas, C. J. Selecting, Acquiring, and Using Small Molecule Libraries for High-Throughput Screening in *Current Protocols in Chemical Biology* (eds Arkin, M.L., Nomura, D., Romesberg, F., Shah, K. & Strano, M. S.) 177–191 (John Wiley & Sons, Inc., 2012). 10.1002/9780470559277.ch110252 PMC4687755 26705509

[83] Kenny, H. A. *et al*. Quantitative high throughput screening using a primary human three-dimensional organotypic culture predicts *in vivo* efficacy. *Nat*. *Commun*. **6** (2015). 10.1038/ncomms7220 PMC4427252 25653139

[84] Gurbanov R, Gozen AG, Severcan F (2018). Rapid classification of heavy metal-exposed freshwater bacteria by infrared spectroscopy coupled with chemometrics using supervised method. Spectrochim. Acta Part A Mol. Biomol. Spectrosc..

[85] Malek, K., Wood, B. R. & Bambery, K. R. FTIR Imaging of Tissues: Techniques and Methods of Analysis in *Optical Spectroscopy and Computational* Methods *in Biology and Medicine* (ed. Baranska, M.) 419–473 (Springer, 2014).

[86] Penha, R. & Hines, J. Using principal component analysis modeling to monitor temperature sensors in a nuclear research reactor. https://pdfs.semanticscholar.org/24d9/3396311e2b915424dc8c69c46ee6e75281a6.pdf (2001).

[87] Valle S, Li W, Qin SJ (1999). Selection of the Number of Principal Components: The Variance of the Reconstruction Error Criterion with a Comparison to Other Methods^†^. Ind. Eng. Chem. Res..

[88] Whelan DR (2014). Detection of an en masse and reversible B- to A-DNA conformational transition in prokaryotes in response to desiccation. J. R. Soc. Interface.

[89] Gurbanov R, Simsek Ozek N, Gozen AG, Severcan F (2015). Quick Discrimination of Heavy Metal Resistant Bacterial Populations Using Infrared Spectroscopy Coupled with Chemometrics. Anal. Chem..

[90] Camo Software As. Multiple Linear Regression. in *The Unscrambler X User Manual* 583–616 (2014).

[91] Morris GM (2009). Software news and updates AutoDock4 and AutoDockTools4: Automated docking with selective receptor flexibility. J. Comput. Chem..

[92] Elrod-Erickson M, Rould MA, Nekludova L, Pabo CO (1996). Zif268 protein-DNA complex refined at 1.6 Å: A model system for understanding zinc finger-DNA interactions. Structure.

[93] O'Boyle Noel M, Banck Michael, James Craig A, Morley Chris, Vandermeersch Tim, Hutchison Geoffrey R (2011). Open Babel: An open chemical toolbox. Journal of Cheminformatics.

[94] Forli S (2016). Computational protein-ligand docking and virtual drug screening with the AutoDock suite. Nat. Protoc..

[95] Huey, R., Morris, G. M. & Forli, S. Using AutoDock 4 and AutoDock Vina with AutoDockTools: A Tutorial. 1–32, http://autodock.scripps.edu/faqs-help/tutorial/using-autodock-4-with-autodocktools/2012_ADTtut.pdf (2012).

[96] Pettersen EF (2004). UCSF Chimera—A Visualization System for Exploratory Research and Analysis. J Comput Chem.

[97] Frisch, M. J. *et al*. Gaussian 09, Revision A.02. *Gaussian*, *Inc*. *Wallingford CT* (2016).

[98] Davis, S. Using the GEOquery Package. https://bioconductor.org/packages/release/bioc/vignettes/GEOquery/inst/doc/GEOquery.html (2014).

[99] Zhijin(Jean) Wu, R. I. Description of gcrma package. https://www.bioconductor.org/packages/devel/bioc/vignettes/gcrma/inst/doc/gcrma2.0.pdf (2018).

[100] hgu133plus2.db. http://bioconductor.org/packages/release/data/annotation/manuals/hgu133plus2.db/man/hgu133plus2.db.pdf (2018).

[101] Package ‘limma’. https://bioconductor.org/packages/release/bioc/manuals/limma/man/limma.pdf (2018).

[102] Package ‘stringr’. https://cran.r-project.org/web/packages/stringr/stringr.pdf (2018).

[103] Venables, W. N., D. M. S. & Team, and the R. C. An Introduction to R. https://cran.r-project.org/doc/manuals/r-release/R-intro.pdf (2018).

