# Supplementary material for: Low dose dimethyl sulfoxide driven gross molecular changes have the potential to interfere with various cellular processes
Source: Sci Rep. 2018 Oct 4;8:14828. doi: 10.1038/s41598-018-33234-z (PMC6172209; doi:10.1038/s41598-018-33234-z)
Supplement: Supplementary file 1 — Supplementary Figures S1 - S11 [file 41598_2018_33234_MOESM1_ESM.pdf]

Supplementary information

Low dose dimethyl sulfoxide driven gross molecular changes have the potential to interfere with various cellular processes

Sinem Tunçer[1, 4], Rafig Gurbanov[2], Ilir Sheraj[1], Ege Solel[1, 5], Okan Esenturk[3], Sreeparna Banerjee[1*]

[1]Department of Biological Sciences, Orta Dogu Teknik Universitesi (ODTU/METU), Ankara, 06800, Turkey.
[2]Department of Molecular Biology and Genetics, Bilecik Şeyh Edebali University, Bilecik, 11230, Turkey.
[3]Department of Chemistry, Orta Dogu Teknik Universitesi (ODTU/METU), Ankara, 06800, Turkey.
[4]Current Address: Vocational School of Health Services, Department of Medical Laboratory Techniques, Bilecik Şeyh Edebali University, Bilecik, 11230, Turkey.
[5]Current Address: Department of Biomedicine, University of Bergen, Bergen, Postbox 7804, N-5020, Norway.
[*]Corresponding author, e-mail: banerjee@metu.edu.

Primary antibodies and dilutions	Sources of primary antibodies	Secondary Antibodies	Source of secondary antibodies and dilutions
Caspase 3 1:300	Santa Cruz Biotechnology, sc-7148	Rabbit	Santa Cruz Biotechnology, sc-2004, 1:2000
Phospho-Rb 1:1000	Cell Signaling Technology, #8516S	Rabbit	Santa Cruz Biotechnology, sc-2004, 1:2000
Cyclin D1 1:1000	Cell Signaling Technology, #2922	Rabbit	Santa Cruz Biotechnology, sc-2004, 1:2000
Cyclin E 1:2000	Merck Millipore, #05-363	Mouse	Santa Cruz Biotechnology, sc-2005, 1:2000
CDK4 1:500	ABCAM, ab68266	Rabbit	Santa Cruz Biotechnology, sc-2004, 1:2000
p21 1:1000	ABCAM, ab109199	Rabbit	Santa Cruz Biotechnology, sc-2004, 1:2000
β-actin 1:4000	Santa Cruz Biotechnology, sc-47778	Mouse	Santa Cruz Biotechnology, sc-2005, 1:2000
GAPDH 1:4000	Santa Cruz Biotechnology, sc-25778	Rabbit	Santa Cruz Biotechnology, sc-2004, 1:2000

Supplementary Table S1. Primary and secondary antibodies used for western blot. A detailed inventory of each primary antibody, and each corresponding secondary antibody used in this study are given, including working dilutions and supporting documentation.

BDNA	Binding Energy (Kcal/mol)	Ligand Efficiency	Inhibition Constant (units/mM)	van der Waals Energy	Electrostatic Energy	ZDNA	Binding Energy (Kcal/mol)	Ligand Efficiency	Inhibition Constant (units/mM)	van der Waals Energy	Electrostatic Energy
**1**	-2.48	-0.6	-15.3	-2	-0.09	**1**	-2.57	-0.64	-13.17	-2.2	-0.36
**2**	-2.48	-0.6	-15.3	-2	-0.1	**2**	-2.75	-0.64	-13.1	-2.41	-0.16
**3**	-2.48	-0.6	-15.3	-2	-0.09	**3**	-2.57	-0.64	-13.14	-2.41	-0.16
**4**	-2.48	-0.6	-15.3	-2	-0.09	**4**	-2.57	-0.64	-13.17	-2.4	-0.17
**5**	-2.48	-0.6	-15.3	-2	-0.09	**5**	-2.57	-0.64	-13.17	-2.4	-0.17
**6**	-2.46	-0.6	-15.6	-2	-0.07	**6**	-2.57	-0.64	-13.14	-2.21	-0.36
**7**	-2.46	-0.6	-15.7	-2	-0.07	**7**	-2.57	-0.64	-13.11	-2.41	-0.16
**8**	-2.46	-0.6	-17.7	-2	-0.06	**8**	-2.56	-0.64	-13.33	-2.18	-0.37
**9**	-2.46	-0.6	-17.7	-2	-0.07	**9**	-2.56	-0.64	-13.22	-2.18	-0.38
**10**	-2.46	-0.6	-15.6	-2	-0.07	**10**	-2.56	-0.64	-13.24	-2.4	-0.16
**11**	-2.46	-0.6	-15.7	-2	-0.07	**11**	-2.56	-0.64	-13.26	-2.4	-0.16
**12**	-2.46	-0.6	-15.7	-2	-0.06	**12**	-2.56	-0.64	-13.22	-2.41	-0.16
**13**	-2.46	-0.6	-15.6	-2	-0.07	**13**	-2.56	-0.64	-13.34	-2.21	-0.35
**14**	-2.46	-0.6	-15.7	-2	-0.07	**14**	-2.56	-0.64	-13.32	-2.39	-0.17
**15**	-2.46	-0.6	-15.6	-2	-0.07	**15**	-2.56	-0.64	-13.32	-2.34	-0.22
**16**	-2.46	-0.6	-15.7	-2	-0.07	**16**	-2.56	-0.64	-13.26	-2.4	-0.16
**17**	-2.46	-0.6	-15.7	-2	-0.11	**17**	-2.56	-0.64	-13.31	-2.33	-0.23
**18**	-2.46	-0.6	-15.7	-2	-0.07	**18**	-2.56	-0.64	-13.34	-2.33	-0.22
**19**	-2.46	-0.6	-15.7	-2	-0.06	**19**	-2.55	-0.64	-13.47	-2.39	-0.16
**20**	-2.46	-0.6	-15.7	-2	-0.06	**20**	-2.55	-0.64	-13.49	-2.38	-0.17
**21**	-2.46	-0.6	-15.7	-2	-0.07	**21**	-2.55	-0.64	-13.4	-2.33	-0.23
**22**	-2.45	-0.6	-15.9	-2	-0.12	**22**	-2.54	-0.64	-13.84	-2.23	-0.3
**23**	-2.45	-0.6	16.07	-2	-0.07	**23**	-2.54	-0.64	-13.73	-2.25	-0.29
**24**	-2.45	-0.6	-15.9	-2	-0.12	**24**	-2.54	-0.64	-13.82	-2.35	-0.18
**25**	-2.45	-0.6	-15.9	-2	-0.12	**25**	-2.54	-0.64	-13.82	-2.22	-0.31
**26**	-2.45	-0.6	-16	-2	-0.13	**26**	-2.54	-0.64	-13.84	-2.35	-0.18
**27**	-2.45	-0.6	-16.6	-2	-0.1	**27**	-2.53	-0.63	-13.97	-2.24	-0.29
**28**	-2.45	-0.6	-15.9	-2	-0.09	**28**	-2.53	-0.63	-13.88	-2.24	-0.3
**29**	-2.42	-0.6	-16.9	-2	-0.02	**29**	-2.53	-0.63	-13.87	-2.31	-0.22
**30**	-2.42	-0.6	-16.9	-2	-0.02	**30**	-2.47	-0.62	-15.49	-2.3	-0.17

Supplementary Table S2. AutoDock energy calculations of DMSO binding to Z- and B-DNA. 30 Calculations were done for each case and the best ligand position was selected according to binding energy. Other parameters include ligand efficiency, inhibition constant, van der Waals and electrostatic energy.

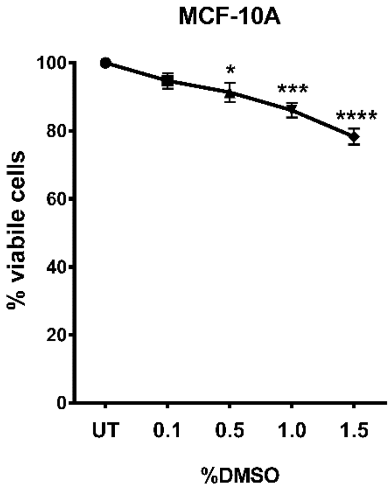

Supplementary Figure S1. DMSO has growth inhibitory effect on MCF-10A cells. After 24 h of incubation with the indicated doses of DMSO, cellular growth was analysed in MCF-10A cells with the MTT assay. The effect of DMSO treatment on cellular growth is expressed as percent viability with respect to untreated (UT) cells. The results from two independent biological replicates each with eight technical replicates are given as mean±SEM. Statistical significance was calculated respect to UT cells by using t test.

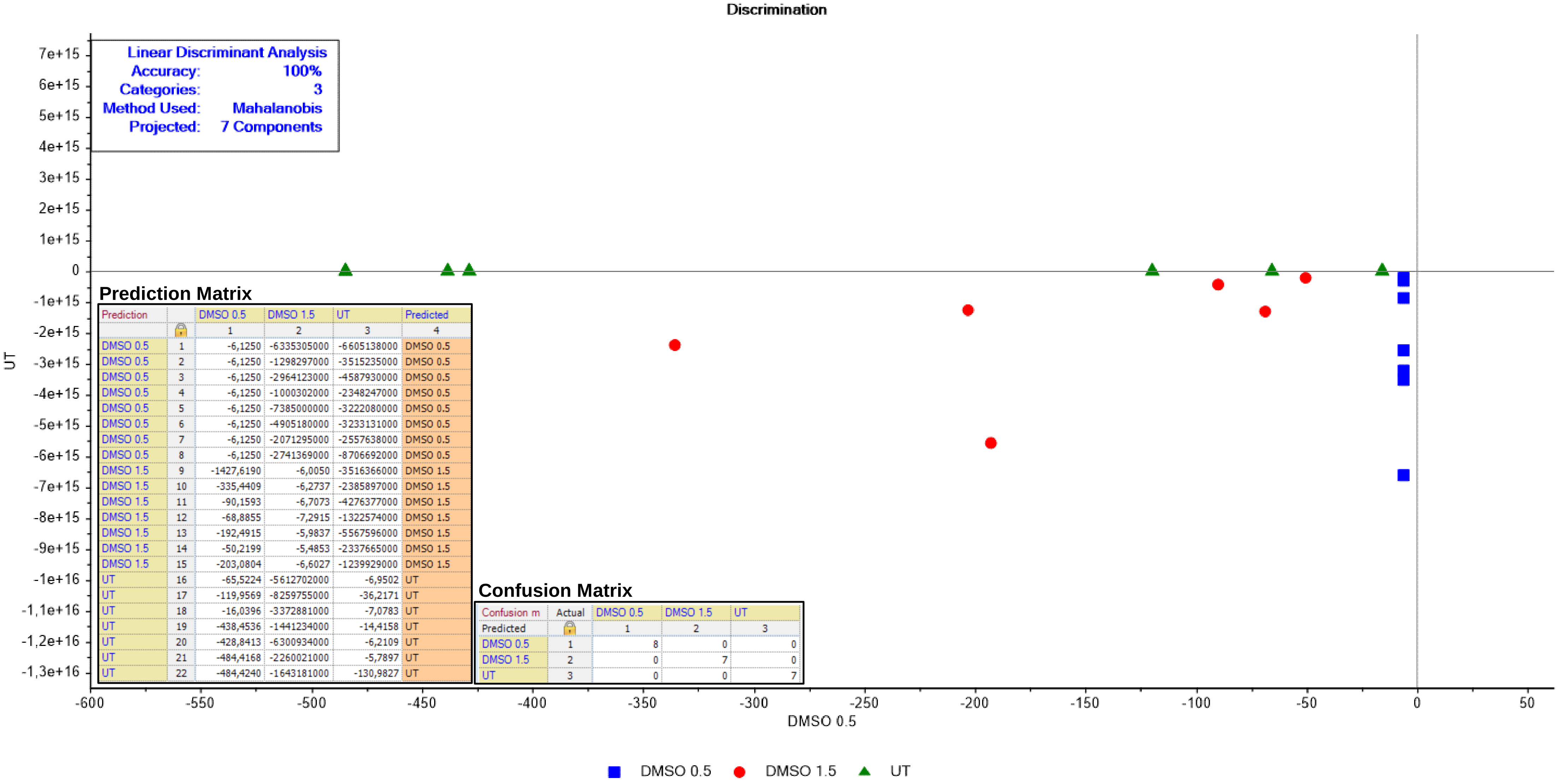

Prediction Matrix

Prediction		DMSO 0.5	DMSO 1.5	UT	Predicted
		1	2	3	4
DMSO 0.5	1	-6,1250	-6335305000	-6605138000	DMSO 0.5
DMSO 0.5	2	-6,1250	-1298297000	-3515235000	DMSO 0.5
DMSO 0.5	3	-6,1250	-2964123000	-4587930000	DMSO 0.5
DMSO 0.5	4	-6,1250	-1000302000	-2348247000	DMSO 0.5
DMSO 0.5	5	-6,1250	-7385000000	-3222080000	DMSO 0.5
DMSO 0.5	6	-6,1250	-4905180000	-3233131000	DMSO 0.5
DMSO 0.5	7	-6,1250	-2071295000	-2557638000	DMSO 0.5
DMSO 0.5	8	-6,1250	-2741369000	-8706692000	DMSO 0.5
DMSO 1.5	9	-1427,6190	-6,0050	-3516366000	DMSO 1.5
DMSO 1.5	10	-335,4409	-6,2737	-2385897000	DMSO 1.5
DMSO 1.5	11	-90,1593	-6,7073	-4276377000	DMSO 1.5
DMSO 1.5	12	-68,8855	-7,2915	-1322574000	DMSO 1.5
DMSO 1.5	13	-192,4915	-5,9837	-5567596000	DMSO 1.5
DMSO 1.5	14	-50,2199	-5,4853	-2337665000	DMSO 1.5
DMSO 1.5	15	-203,0804	-6,6027	-1239929000	DMSO 1.5
UT	16	-65,5224	-5612702000	-6,9502	UT
UT	17	-119,9569	-8259755000	-36,2171	UT
UT	18	-16,0396	-3372881000	-7,0783	UT
UT	19	-438,4536	-1441234000	-14,4158	UT
UT	20	-428,8413	-6300934000	-6,2109	UT
UT	21	-484,4168	-2260021000	-5,7897	UT
UT	22	-484,4240	-1643181000	-130,9827	UT

Confusion Matrix

Confusion m	Actual	DMSO 0.5	DMSO 1.5	UT
Predicted		1	2	3
DMSO 0.5	1	8	0	0
DMSO 1.5	2	0	7	0
UT	3	0	0	7

Supplementary Figure S2. LDA discrimination plot, and prediction and confusion matrices for DMSO treated (0.5% and 1.5% v/v) and untreated (UT) HCT-116 cells in 4000-650 cm^{-1} spectral region.

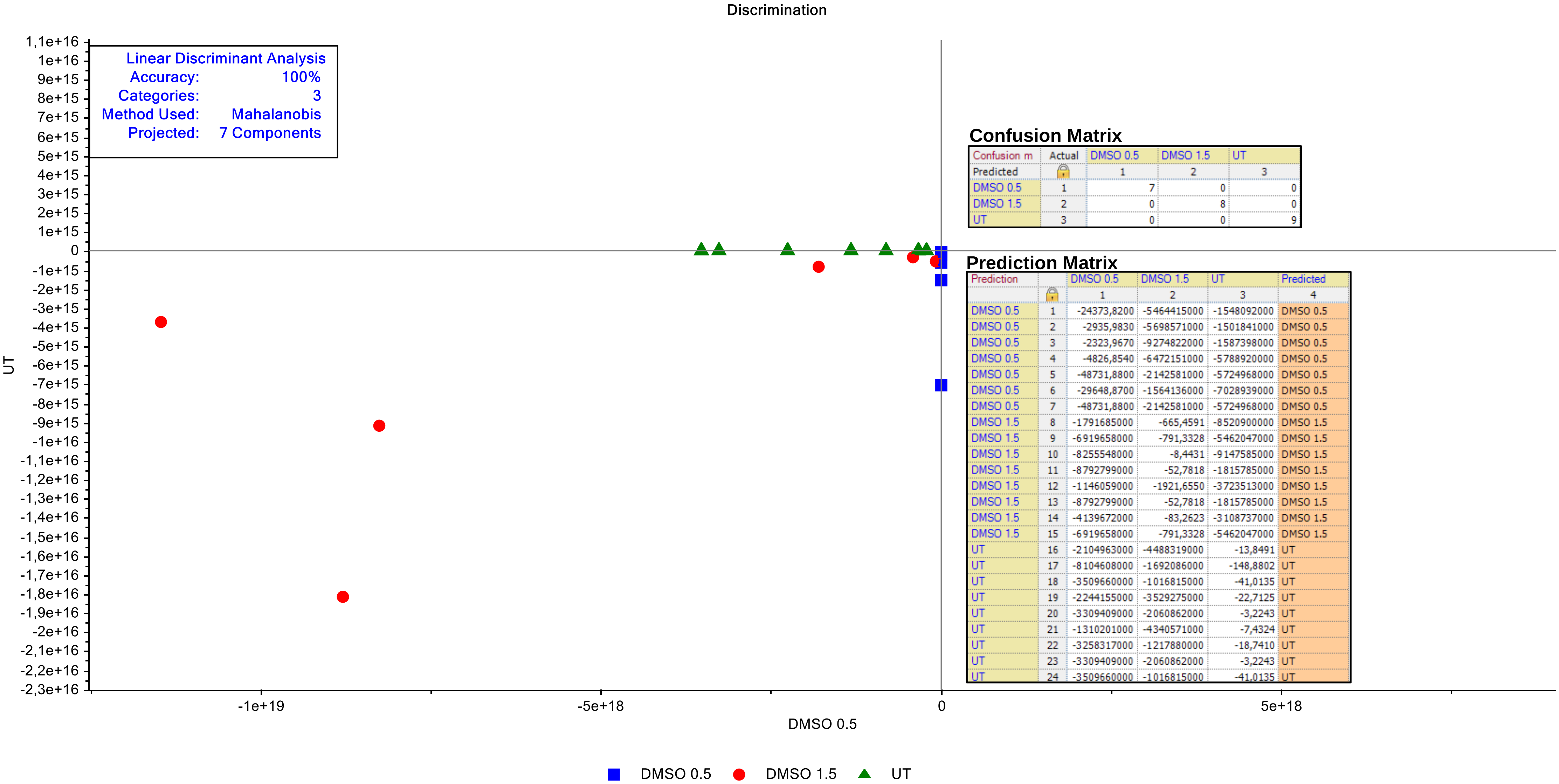

Confusion Matrix

Confusion m	Actual	DMSO 0.5	DMSO 1.5	UT
Predicted		1	2	3
DMSO 0.5	1	7	0	0
DMSO 1.5	2	0	8	0
UT	3	0	0	9

Prediction Matrix

Prediction		DMSO 0.5	DMSO 1.5	UT	Predicted
		1	2	3	4
DMSO 0.5	1	-24373,8200	-5464415000	-1548092000	DMSO 0.5
DMSO 0.5	2	-2935,9830	-5698571000	-1501841000	DMSO 0.5
DMSO 0.5	3	-2323,9670	-9274822000	-1587398000	DMSO 0.5
DMSO 0.5	4	-4826,8540	-6472151000	-5788920000	DMSO 0.5
DMSO 0.5	5	-48731,8800	-2142581000	-5724968000	DMSO 0.5
DMSO 0.5	6	-29648,8700	-1564136000	-7028939000	DMSO 0.5
DMSO 0.5	7	-48731,8800	-2142581000	-5724968000	DMSO 0.5
DMSO 1.5	8	-1791685000	-665,4591	-8520900000	DMSO 1.5
DMSO 1.5	9	-6919658000	-791,3328	-5462047000	DMSO 1.5
DMSO 1.5	10	-8255548000	-8,4431	-9147585000	DMSO 1.5
DMSO 1.5	11	-8792799000	-52,7818	-1815785000	DMSO 1.5
DMSO 1.5	12	-1146059000	-1921,6550	-3723513000	DMSO 1.5
DMSO 1.5	13	-8792799000	-52,7818	-1815785000	DMSO 1.5
DMSO 1.5	14	-4139672000	-83,2623	-3108737000	DMSO 1.5
DMSO 1.5	15	-6919658000	-791,3328	-5462047000	DMSO 1.5
UT	16	-2104963000	-4488319000	-13,8491	UT
UT	17	-8104608000	-1692086000	-148,8802	UT
UT	18	-3509660000	-1016815000	-41,0135	UT
UT	19	-2244155000	-3529275000	-22,7125	UT
UT	20	-3309409000	-2060862000	-3,2243	UT
UT	21	-1310201000	-4340571000	-7,4324	UT
UT	22	-3258317000	-1217880000	-18,7410	UT
UT	23	-3309409000	-2060862000	-3,2243	UT
UT	24	-3509660000	-1016815000	-41,0135	UT

Supplementary Figure S3. LDA discrimination plot, and prediction and confusion matrices for DMSO treated (0.5% and 1.5% v/v) and untreated (UT) SW-480 cells in 4000-650 cm^{-1} spectral region.

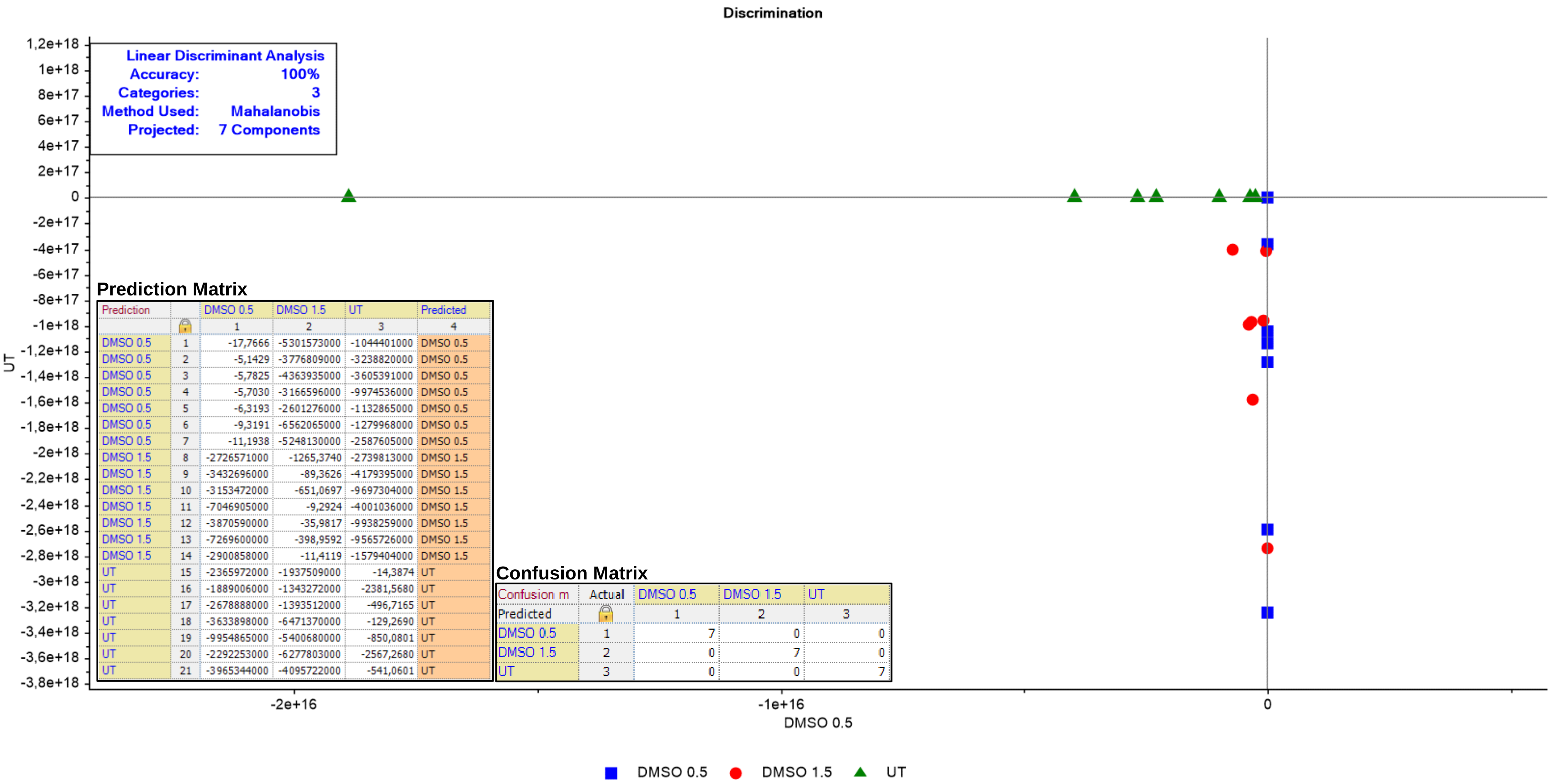

Prediction Matrix

Prediction		DMSO 0.5	DMSO 1.5	UT	Predicted
		1	2	3	4
DMSO 0.5	1	-17,7666	-5301573000	-1044401000	DMSO 0.5
DMSO 0.5	2	-5,1429	-3776809000	-3238820000	DMSO 0.5
DMSO 0.5	3	-5,7825	-4363935000	-3605391000	DMSO 0.5
DMSO 0.5	4	-5,7030	-3166596000	-9974536000	DMSO 0.5
DMSO 0.5	5	-6,3193	-2601276000	-1132865000	DMSO 0.5
DMSO 0.5	6	-9,3191	-6562065000	-1279968000	DMSO 0.5
DMSO 0.5	7	-11,1938	-5248130000	-2587605000	DMSO 0.5
DMSO 1.5	8	-2726571000	-1265,3740	-2739813000	DMSO 1.5
DMSO 1.5	9	-3432696000	-89,3626	-4179395000	DMSO 1.5
DMSO 1.5	10	-3153472000	-651,0697	-9697304000	DMSO 1.5
DMSO 1.5	11	-7046905000	-9,2924	-4001036000	DMSO 1.5
DMSO 1.5	12	-3870590000	-35,9817	-9938259000	DMSO 1.5
DMSO 1.5	13	-7269600000	-398,9592	-9565726000	DMSO 1.5
DMSO 1.5	14	-2900858000	-11,4119	-1579404000	DMSO 1.5
UT	15	-2365972000	-1937509000	-14,3874	UT
UT	16	-1889006000	-1343272000	-2381,5680	UT
UT	17	-2678888000	-1393512000	-496,7165	UT
UT	18	-3633898000	-6471370000	-129,2690	UT
UT	19	-9954865000	-5400680000	-850,0801	UT
UT	20	-2292253000	-6277803000	-2567,2680	UT
UT	21	-3965344000	-4095722000	-541,0601	UT

Confusion Matrix

Confusion m	Actual	DMSO 0.5	DMSO 1.5	UT
Predicted		1	2	3
DMSO 0.5	1	7	0	0
DMSO 1.5	2	0	7	0
UT	3	0	0	7

Supplementary Figure S4. LDA discrimination plot, and prediction and confusion matrices for DMSO treated (0.5% and 1.5% v/v) and untreated (UT) MCF-10A cells in 4000-650 cm^{-1} spectral region.

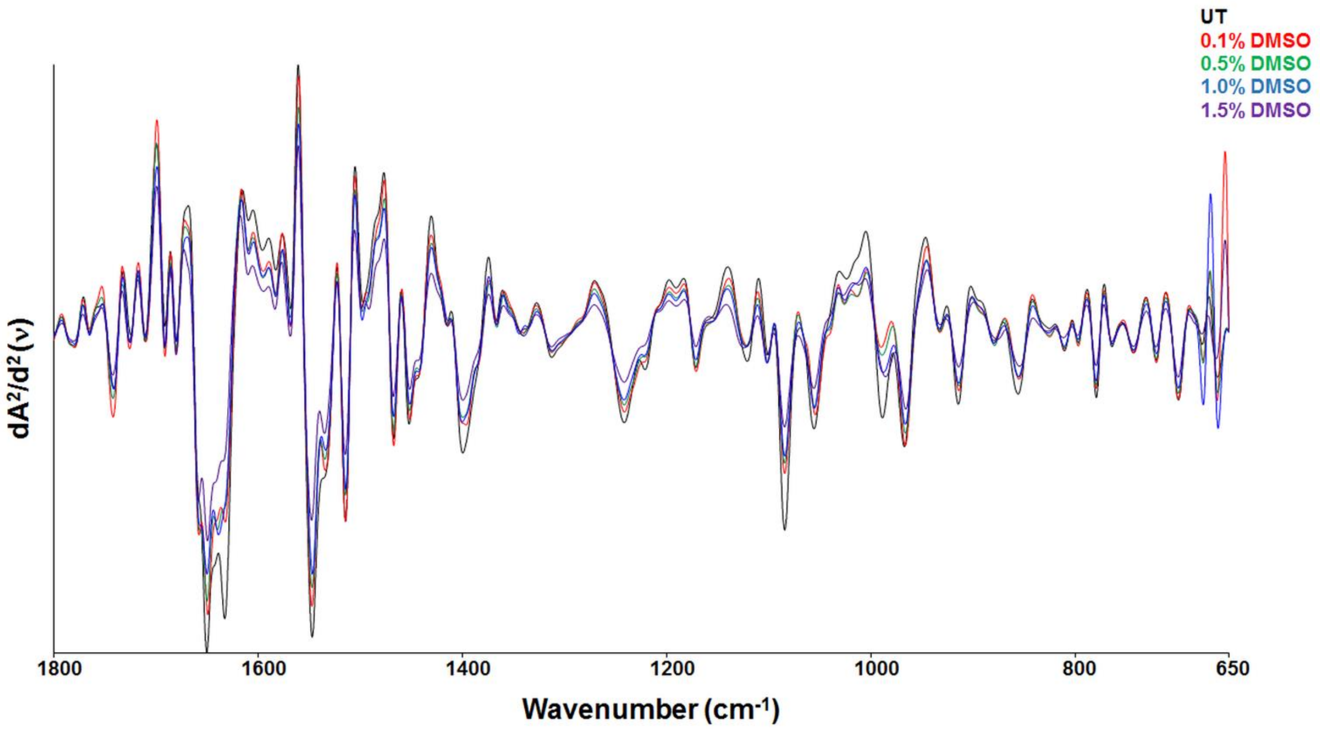

Supplementary Figure S5. The representative IR spectra of DMSO treated and untreated cells. The second derivative and vector-normalized IR spectra of DMSO treated (0.1%-1.5%, v/v) and untreated (UT) HCT-116 cells in fingerprint (1800-650 cm^{-1}) spectral region.

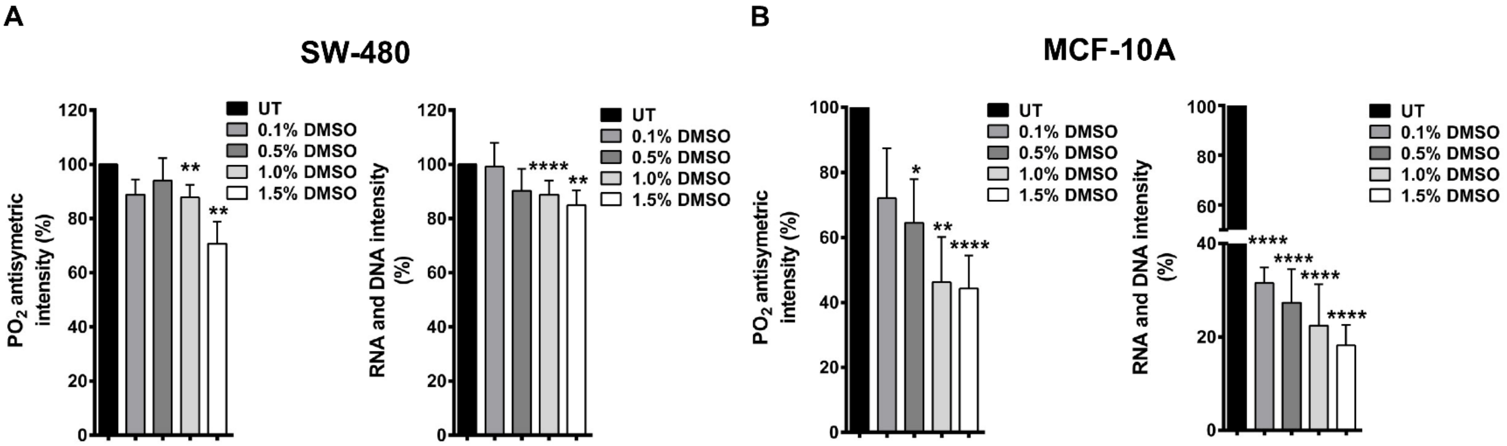

Supplementary Figure S6. DMSO decreases cellular nucleic acid content in SW-480 and MCF-10A cells. (PO_2 antisymmetric values (% band intensity), and Ribose ring vibrations of total RNA and DNA values (% band intensity) for DMSO treated and UT **(A)** SW-480 and **(B)** MCF-10A cells are shown. Results are presented as mean±SEM, and statistical significance was calculated with respect to UT cells by using t test.

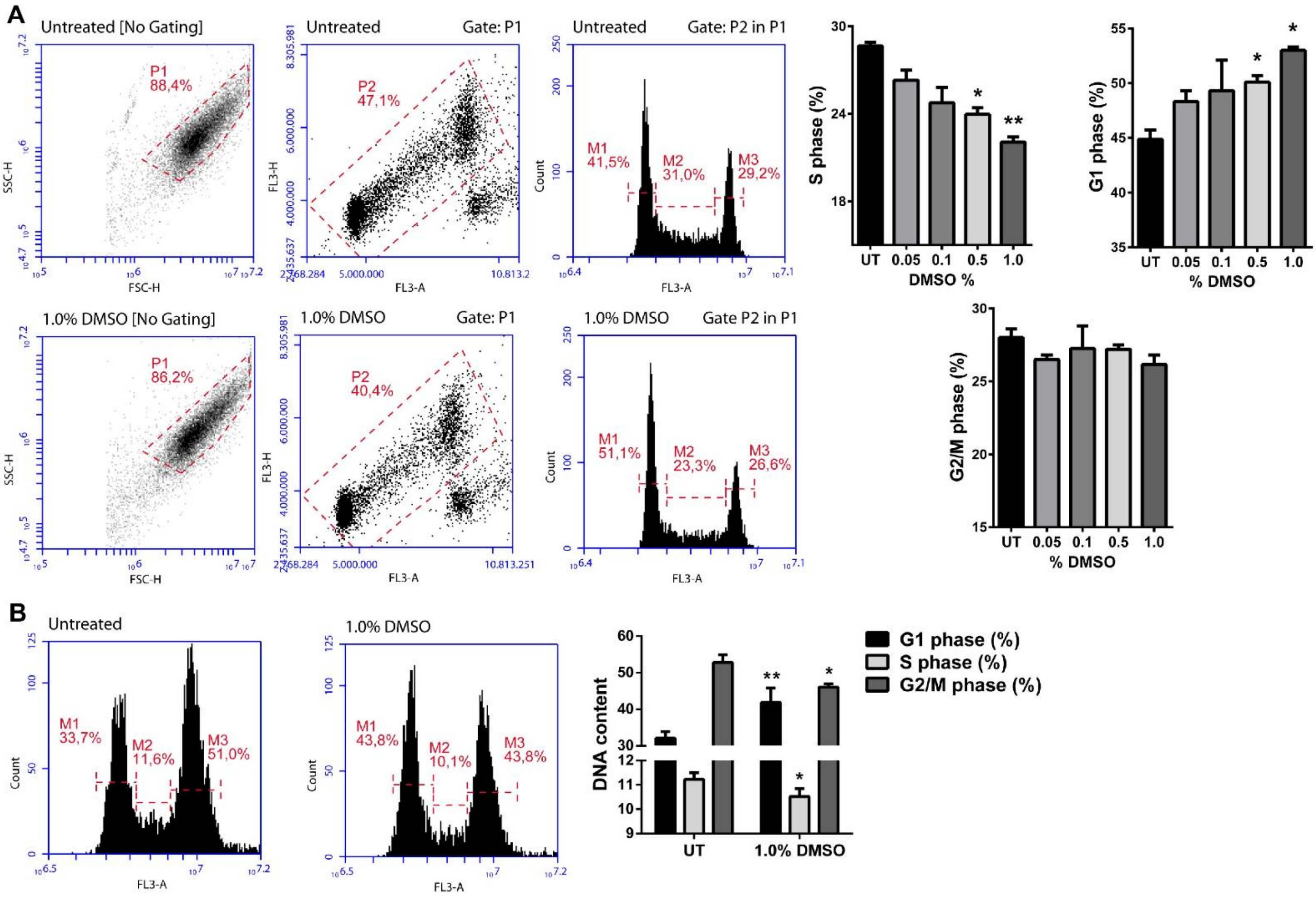

Supplementary Figure S7. DMSO treatment increases the number of G1 phase HCT-116 and SW-480 cells. (A) Proliferating HCT-116 cells were treated with 0.05-1.0% DMSO for 24 h. Percentages of G1, S and G2/M phase cells for DMSO treated and untreated (UT) cells are shown as separate graphs. The results are given as mean±SEM of three technical replicates. t test was used for comparison of the results with respect to UT cells. Representative images for the scatter plots showing cell populations, and the histograms showing DNA content frequencies are given. FL3H versus FL3A gate was used to exclude the majority of cell doublets and cell clumps from the analyses. **(B)** Representative histograms show cell cycle phase distribution in 1.0% DMSO treated and untreated (UT) SW-480 cells. Percentage of the phases are shown as a separate graph where the results of three technical replicates are given as mean±SEM. t test was used for the analysis of the results with respect to UT cells.

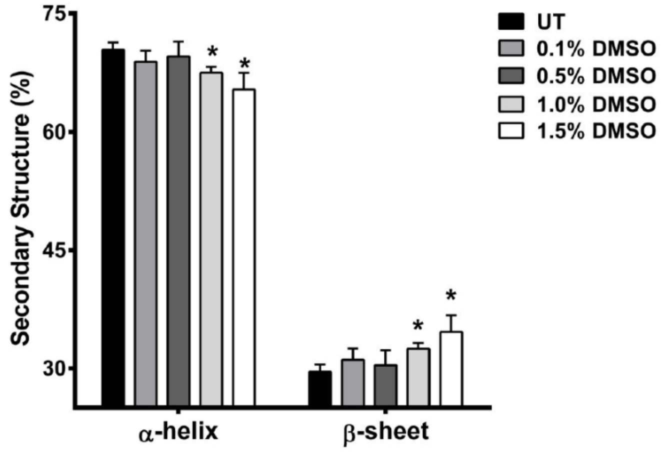

Supplementary Figure S8. Protein secondary structures in SW-480 cells are affected by DMSO treatment. The band intensities (%) of total α-helix and β-sheet structures in DMSO treated and untreated (UT) SW-480 cells are shown. The results are shown as mean±SEM, and the degree of significance was calculated with respect to UT cells by using t test.

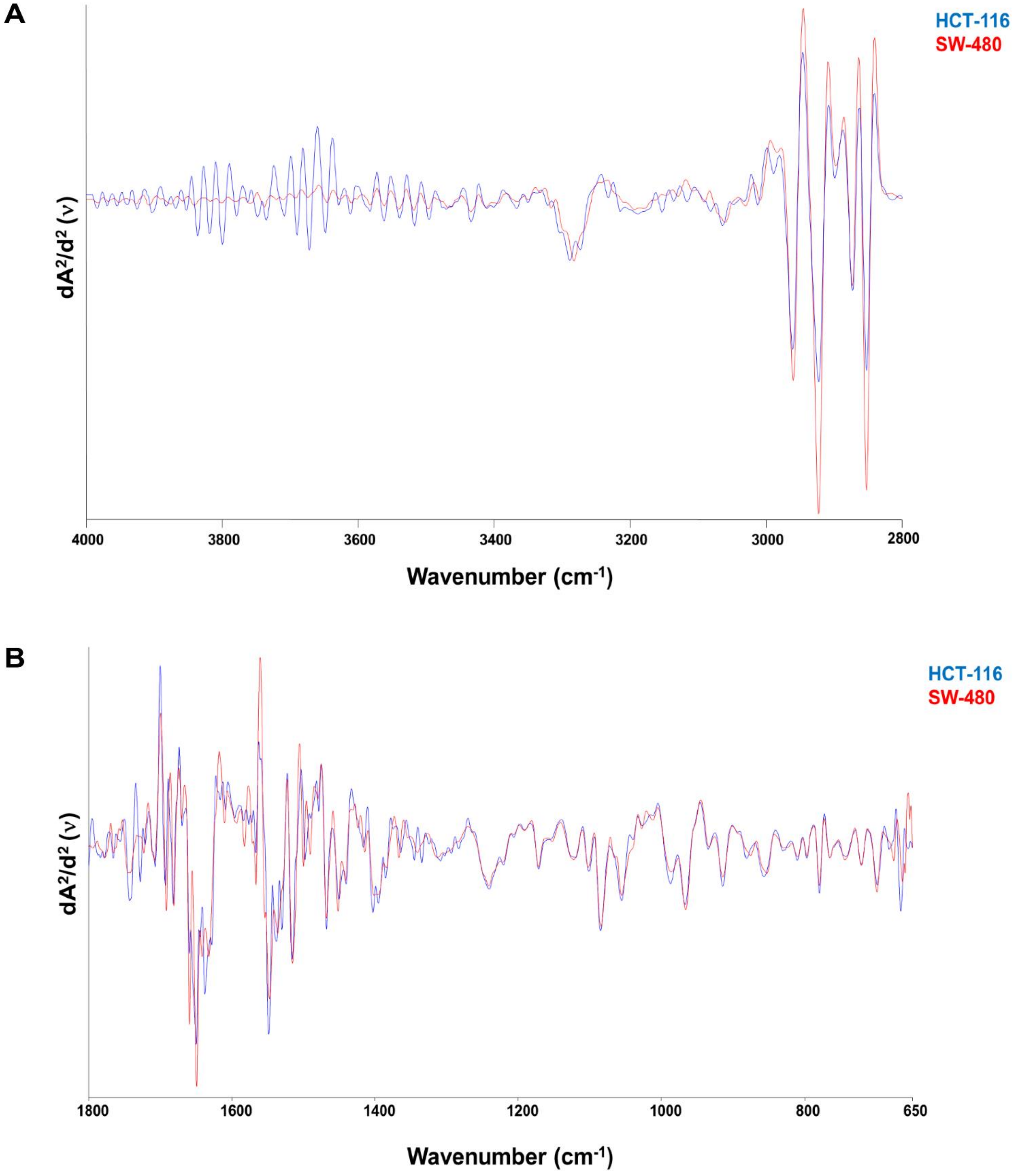

Supplementary Figure S9. Spectral differences between HCT-116 and SW-480 cells. Representative second derivative and vector-normalized IR spectra of HCT-116 cells and SW-480 cells in **(A)** lipid (4000-2800 cm^{-1}) and **(B)** fingerprint (1800-650 cm^{-1}) spectral regions.

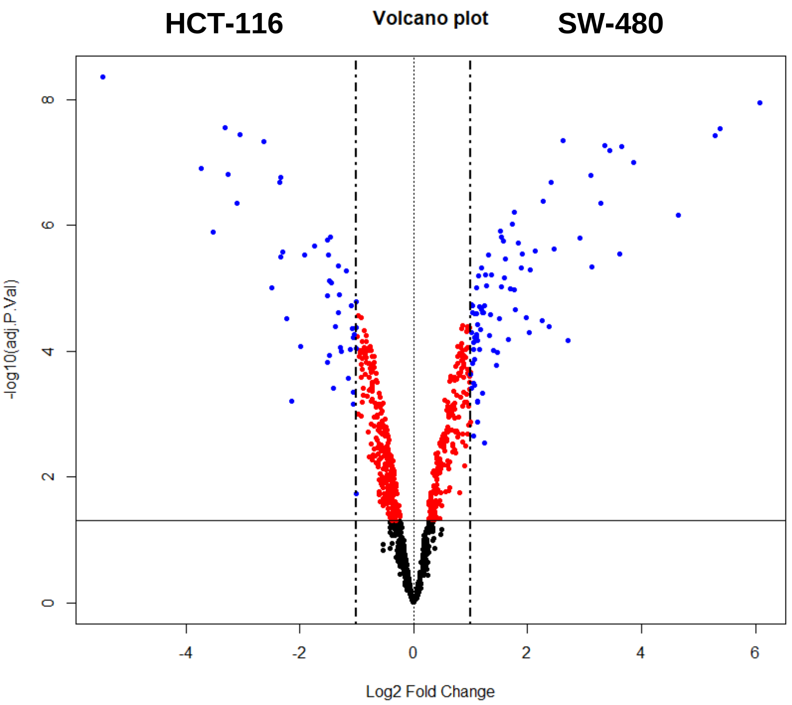

Supplementary Figure S10. Volcano plot showing the differential expression of genes related to the Gene Ontology term LIPID_METABOLISM (GO:0006629) in HCT-116 and SW-480 cells. Genes related to the Gene Ontology (GO) term LIPID_METABOLISM (GO:0006629) were extracted (1039 probes representing more than 500 genes). Based on selection criteria of adjusted p-value < 0.05 and fold change of 1, 130 probes representing 98 genes were found to be differentially regulated in SW-480 and HCT-116 cells. Of these, 84 probes (representing 61 genes) were upregulated in SW-480 and 46 probes (representing 37 genes) were upregulated in HCT-116 cells.

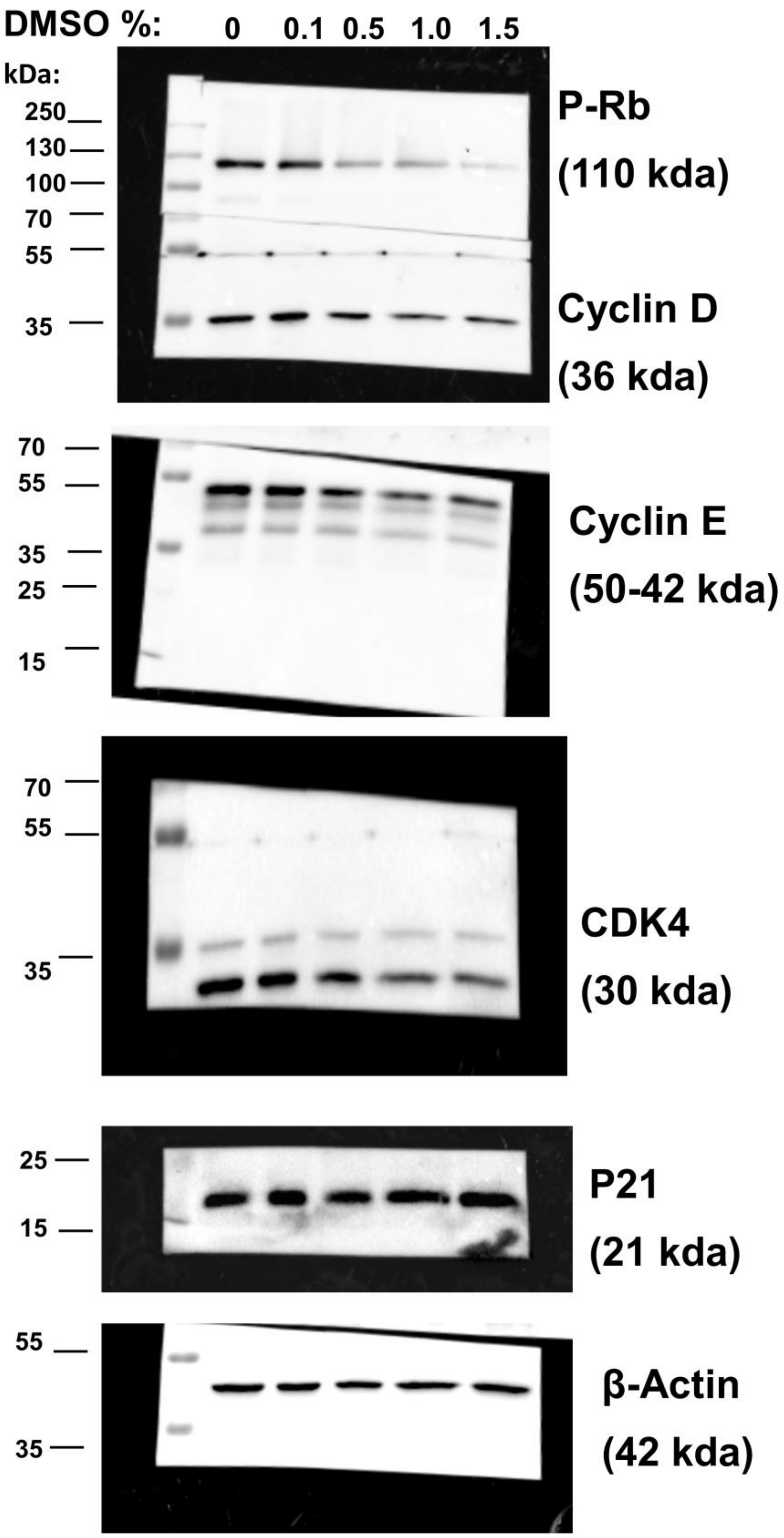

Supplementary Figure S11. Full-length images of Western blots.
